# Abundance of mobile genetic elements in an *Acinetobacter lwoffii* strain isolated from Transylvanian honey sample

**DOI:** 10.1038/s41598-020-59938-9

**Published:** 2020-02-19

**Authors:** Alexandra Veress, Tibor Nagy, Tímea Wilk, János Kömüves, Ferenc Olasz, János Kiss

**Affiliations:** Department of Genetics, Agricultural Biotechnology Institute, National Agricultural Research and Innovation Centre, Gödöllő, H-2100 Hungary

**Keywords:** Bacterial genetics, Bacterial genomics, Food microbiology

## Abstract

Based on phylogenetic analyses, strain M2a isolated from honey, an unexpected source of acinetobacters, was classified as *Acinetobacter lwoffii*. The genome of this strain is strikingly crowded with mobile genetic elements. It harbours more than 250 IS elements of 15 IS-families, several unit and compound transposons and 15 different plasmids. These IS elements, including 30 newly identified ones, could be classified into at least 53 IS species. Regarding the plasmids, 13 of the 15 belong to the Rep-3 superfamily and only one plasmid, belonging to the “Low-GC” family, possesses a seemingly complete conjugative system. The other plasmids, with one exception, have a mobilization region of common pattern, consisting of the divergent *mobA/mobL*-family and *mobS-*, *mobC-* or *traD-*like genes separated by an *oriT-*like sequence. Although two plasmids of M2a are almost identical to those of *A. lwoffi* strains isolated from gold mine or Pleistocene sediments, most of them have no close relatives. The presence of numerous plasmid-borne and chromosomal metal resistance determinants suggests that M2a previously has also evolved in a metal-polluted environment. The numerous, possibly transferable, plasmids and the outstanding number of transposable elements may reflect the high potential of M2a for rapid evolution.

## Introduction

*Acinetobacter* genus belong to the γ-Proteobacteria, *Pseudomonadales* order and *Moraxellaceae* family and includes aerobic, Gram-negative, catalase-positive and oxidase-negative coccobacilli^1,2^. The genus has undergone drastic changes before the proposal of Baumann *et al*.^3^ was accepted by the subcommittee on *Moraxella* and allied bacteria^4^. This proposal limited the genus to oxidase-negative strains, and currently includes ca. 60 validly named species (LPSN, http://www.bacterio.net/-allnamesac.html)^5^. The taxonomy of the genus relies on classical microbial^6^ and diverse biochemical and molecular methods. Over the past decades a variety of methods have been used to classify and identify *Acinetobacter* species, such as DNA-DNA hybridization, AFLP analysis^7^, amplified ribosomal DNA restriction analysis^8,9^, sequence analysis of 16S rDNA and several housekeeping genes (*gyrB*, *rpoB*, *cpn60*, *fusA*, *gltA*, *pyrG*, *recA*, *rplB*)^10^, MALDI-TOF mass spectrometry and evaluation of genomic data^2^. Descriptions of novel species are generally accompanied by a comprehensive set of physiological and nutritional tests, originally developed by^11^ and later extended by Nemec *et al*., e.g.^12–14^.

*Acinetobacter* includes species of different life-styles, from free-living saprophytes to human- and animal-pathogens^2,15^. *Acinetobacter* species occur in diverse natural and artificial environments such as forest and agricultural soils, animal and human skin and gut, fresh- and seawater, or even sewage and activated sludge^1^. Some of them are able to tolerate extreme conditions, for instance low temperature, hydrocarbon-contaminated sites and high osmotic conditions. Despite their high prevalence in most environments, the distribution and ecological roles of *Acinetobacter* species, apart from pathogenic and nosocomial species with clinical importance, are poorly explored^16^. The most studied *Acinetobacter* species is the human pathogen *A. baumannii*^17,18^, which have attracted exceptional attention because of its pathogenicity and multiresistance^19^. Less information is available on non-*baumannii* acinetobacters^15^, living in a wide range of environments including habitats contaminated with heavy metals^20^ or oil^21,22^, cold habitats^23,24^ or high osmotic environments, such as saltern ponds^25^ or floral nectar^26^. To our knowledge, there are no reports of *Acinetobacter* spp. isolated from honey^27^, however there are reports on *Acinetobacter* spp. in the honey bee gut^28^. *Acinetobacter* was suggested as a model organism in the environmental microbiology and pathogenesis^2,29,30^ due to its ecological and clinical importance, the utilization of various kinds of carbon sources and sufficient growth on simple media and the environmental characteristics, which substantially differ from those of the most common enteric model organism, *E. coli*. Several *Acinetobacter* spp. have been regarded as potentially important microorganisms in both environmental and biotechnological applications like bioremediation of soil and water, or production of “bioproducts” (polysaccharides, polyesters, enzymes)^31^.

Mobile genetic elements (MGEs) often harbour various kinds of genes endowing their hosts with resistance to antibiotics and heavy metals, or beneficial traits like virulence or metabolism of unusual substrates^32–34^. Well-known vehicles of such genes are plasmids often capable to transfer horizontally, even between unrelated bacterial species by conjugation or natural transformation. Plasmids therefore play an important role in the evolution and adaptation of bacteria. Even though many resistance genes are not located on the resistance islands but are scattered over the genomes, the genomic resistance islands and plasmids are key players in the emergence of antibiotic resistant *Acinetobacter* strains, which represent a significant health threat e.g. the nosocomial pathogen *A. baumannii*^19,35–37^. More frequent occurrence of plasmids was observed in 75 clinical *Acinetobacter* isolates classified into four non-*baumannii* species and three different multiresistance patterns. Plasmid DNA fingerprinting showed that >84% of these strains contained up to 15 plasmids^38^. In a comparative study, *A. lwoffi* isolates were found to carry more plasmids (≤20) than *A. anitratus* (≤8)^39^. Resistance genes are often associated with transposons and integrons^19,35,40–43^, in addition non-integron cassette streptomycin/spectinomycin resistance gene *aadA27* was also observed in plasmids identified in ancient (isolated from permafrost) and recent environmental *Acinetobacter* isolates^44^.

In this study we describe an *Acinetobacter lwoffii* strain named M2a that was isolated from a Transylvanian honey sample and proved to carry an outstanding number of MGEs. The aim of this project was to investigate the bacterial community of honey and the intestinal tract of honeybees derived from a nearly natural rural meadow, and to isolate *Lactobacilli* or other species that might have probiotic traits. The different stains obtained were classified based on phylogenetic analysis of their 16S rRNA gene, and their plasmid content was also examined. Among these isolates, M2a appeared to be interesting as *Acinetobacter* spp. are rarely isolated from sugar-rich environments and the preliminary examination suggested that the strain carry numerous, possibly undescribed, plasmids.

## Results and Discussion

### Isolation and characterization of *Acinetobacter* strain M2a

Strain M2a was isolated from a honey sample derived from a nearly natural meadow in Transylvania together with many other yet uncharacterized *Acinetobacter*, *Lactobacillus, Lysinibacter*, *Saccharibacter*^45^ and *Sphingobacterium*^46^ strains that could grow under conditions favourable mostly for lactobacilli. Thus, M2a was isolated from an MRS + CaCO_3_ agar plate incubated under CO_2_-enriched condition at 35 °C for 48 h. It proved to be catalase-positive whilst negative in methyl red, Voges-Proskauer, indole, citrate utilization, urease and oxidase tests. When grown on TSI agar, the strain proved to be a glucose, lactose and sucrose non-fermenter, and did not produce detectable amount of H_2_S or other gases. Optimal growth occurred at 30 °C on LB agar, while slower growth was observed at 37 °C, and no growth occurred at 44 °C.

BLAST search using the PCR-amplified 16S rDNA as a query sequence suggested that M2a can be classified as an *Acinetobacter* sp.. The preliminary analysis of its plasmid content indicated that the strain carries multiple small and medium size (<20 kb) plasmids (Suppl. Fig. S1). Due to the fact that *Acinetobacter* strains have rarely been isolated from sugar-rich environments like honey^28^ and to the diverse plasmid content found in M2a, we decided to investigate its genome by WGS.

#### The whole genome sequence

3.6 million Illumina MiSeq reads representing ca. 76× coverage of the whole genome were *de novo* assembled using A5-miseq and the resulting 277 scaffolds (average length: 13053 bp, median: 5605 bp) were annotated by the RAST server. 3637 annotated genes, 153 tRNAs and 36 rRNAs were identified in the scaffolds, whose total length was 3 615 619 bp with 40.44% GC-content as reported for acinetobacters^2^. BLAST searches in the GenBank database with the 277 scaffold sequences indicated that of 168 contained chromosomal sequences, 57 showed at least partial homology to known plasmids, while the remaining 52 scaffolds represented different IS element sequences (Suppl. data 1).

#### Phylogenetic analysis

**Figure 1:**
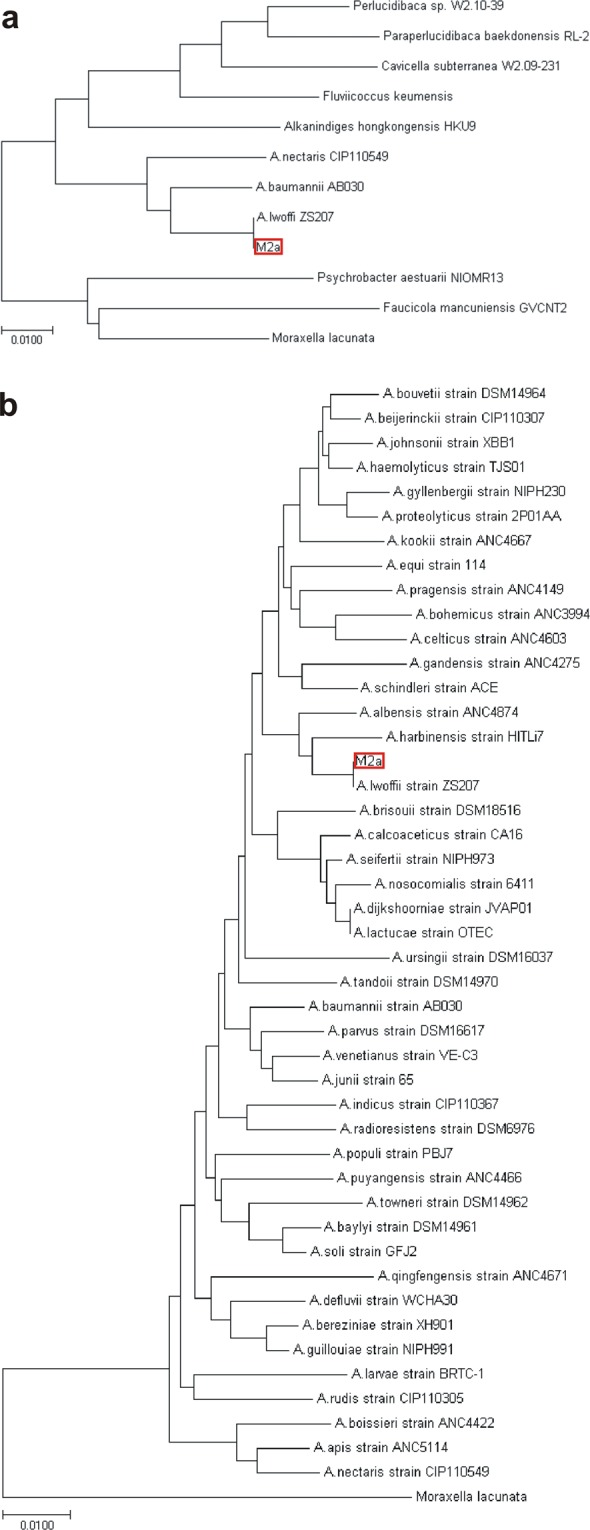
Phylogenetic relationship of strain M2a. (**a**) Phylogenetic tree for the 16S rDNA sequences of representative species from genera of *Moraxellaceae* including strain M2a. (**b**) Phylogenetic tree for the 16S rDNA sequences of 44 species of the genus *Acinetobacter* including strain M2a. The 16S rDNA sequence of *Moraxella lacunata* was included as outgroup. Trees were generated using the neighbour-joining method. Bar, 0.01 changes per nucleotide position.

Phylogenetic relationship of M2a was determined by analyzing the 16S rRNA gene and housekeeping genes *rpoB*, *gyrB* and *recA* (Suppl. data 2). The phylogenetic tree based on the 16S rDNA sequences of M2a and representative members of the family *Moraxellaceae* (Suppl. Table S1a) indicated that our isolate belongs to the *Acinetobacter* genus (Fig. 1a). For more exact classification, WGSs of 44 *Acinetobacter* species with validated names (Suppl. Table S1b) were downloaded from public databases and phylogenetic trees were generated for their 16S rDNA (Fig. 1b), *rpoB*, *gyrB* and *recA* genes (Suppl. Fig. S2). *A. lwoffii* was found to be the closest relative of our isolate and this was also supported by analyses of further housekeeping genes, such as *dnaJ*, *groEL* and *gyrA* (data not shown). A recent study revised the taxonomy of strains formerly classified as *A. lwoffii*, genospecies GS8 and GS9, and created a new species, *A. pseudolwoffii* for group GS8^14^. To decide which species M2a belongs to, an *rpoB*-based analysis was carried out for 13*A. lwoffii* and 13*A. pseudolwoffii* strains as described previously by^14^. The *rpoB* tree confirmed that M2a is a strain of *A. lwoffii* (Suppl. Fig. S3).

#### Antibiotic and metal resistance determinants

Antibiotic susceptibility tests indicated that M2a had low level ampicillin resistance (<50 μg/ml Ap), but was sensitive for antibiotics such as ciprofloxacin, chloramphenicol, erythromycin, florphenicol, gentamycin, kanamycin, nalidixic acid, neomycin, rifampicin, spectinomycin, streptomycin, tetracycline and zeocin in the concentrations used in standard microbial methods (Table 1). Although the MIC value of M2a was determined as 100 μg/ml for ampicillin (Ap), the 100× dilution of the tested overnight culture (containing ca. 1.25 × 10^4^ cells) gave only a few colonies on LB agar + 100 μg/ml Ap. Additionally, the CFU was 2 orders of magnitude lower in the presence of only 50 μg/ml Ap than in absence of Ap (1.0 × 10^6^ vs. 2.5 × 10^8^/ml, respectively), indicating that less than 0.5% of M2a cells could form colonies on LB agar + 50 μg/ml Ap. A similar gradual increase of CFU was observed with decreasing concentrations of ciprofloxacin and rifampicin.

**Table 1:** MIC values for M2a.

Antibiotics	μg/ml	Metal ions	mM
Ampicillin	100	Hg^2+^	>0.075
Ciprofloxacin	0.75	Cd^2+^	0.2
Chloramphenicol	10	Co^2+^	0.8
Florfenicol	<10	Cu^2+^	2.7
Erythromycin	<25	Zn^2+^	1.6
Gentamycin	<2		
Kanamycin	<10		
Neomycin	<10		
Nalidixic acid	<5		
Rifampicin	75		
Streptomycin	<10		
Spectinomycin	20		
Tetracycline	<2		
Zeocin	<50		

After the susceptibility test, the WGS of M2a was searched for potential resistance genes using public databases CARD, MEGARes and the ARG-ANNOT server. 7 unspecific AR loci and a gene for carbapenem-hydrolyzing class D β-lactamase OXA-283 (OXA-134 family) were identified in the chromosome (Table 2). BLAST searches revealed that *bla* OXA-283 of M2a is identical to *bla* OXA of *Acinetobacter* sp. CIP A162 (NG_049581) and also occurs in many *A. lwoffii* strains. It does not carry the 9-bp deletion characteristic for several strains of *A. lwoffii*/genomic species 9^47^ and shows 93–99% similarity to its homologs in *A. lwoffii*. Thus, the presence of the chromosomal OXA-134 gene further supports the results of the phylogenetic analyses, which advocated that M2a should be designated as *A. lwoffii*. The best match was found with *A. lwoffii* strain ZS207, where the orthologous gene carries only 3 SNPs (causing no amino acid changes) and the 400 bp upstream regions are also identical. Despite the apparently intact coding sequence and upstream region (probably containing the promoter), M2a proved to be sensitive to >50 μg/ml ampicillin. Disc tests were performed with new generation carbapenems cefotaxime and ceftazidime, against which the OXA-134 family β-lactamases are efficient enzymes^48^. The inhibitory zone diameter was >26 mm for each drug indicating that M2a is sensitive to these antibiotics, similarly to other *A. lwoffii* strains carrying chromosomal OXA-134 genes^48^.

**Table 2:** Antibiotic and metal resistance determinants of M2a.

Annotated resistance gene(s)	Localization in WGS
**Antibiotic resistance**	
Quaternary ammonium compound resistance protein gene *sugE*	sc_6
ABC-type multidrug transport system	sc_10
*acrAB*-like RND multidrug efflux transporter system	sc_108
Bcr/CflA family multidrug resistance transporter system	sc_44
*cmeABC*-like RND-family multidrug efflux system	sc_112
Macrolide-specific efflux RND transporter protein genes *macAM, cmeC*	sc_3
Macrolide-specific efflux RND transporter protein gene *macA*	sc_76
Class D β-lactamase OXA-283 (OXA-134 family)	sc_61
**Metal resistance**	
Arsenate reductase	sc_4
Arsenic resistance operon	sc_56
Chromate efflux transporter protein gene *chrA*	sc_44
Copper resistance protein gene *copB*, multicopper oxidase gene	sc_4
Copper resistance protein genes (*copB*, oxidase, *cusR*)	sc_93
Copper resistance protein genes *copCD*	sc_164
Czc-family cobalt/zinc/cadmium efflux RND transporter system genes *czcABCD*	sc_65
Czc-family cobalt/zinc/cadmium resistance protein gene *czcD*	sc_13,
Czc-family cobalt/zinc/cadmium resistance protein gene *czcD*	sc_25,
Czc-family cobalt/zinc/cadmium resistance protein gene *czcD*	sc_184
Lead, cadmium, zinc and mercury transporting ATPase	sc_93
Mercuric resistance module (*mer*) associated with Tn*As2*	sc_65
Tellurium resistance protein gene *tehB*	sc_11
Tellurium resistance protein genes *klaAB*	sc_13
Tellurite resistance protein-related protein gene	sc_49

The M2a genome proved to also carry many metal resistance determinants (Table 2), thus the resistance of M2a for several heavy metal ions was assayed. MIC values were similar to those of *A. lwoffii* strains isolated from Kolyma Lowland permafrost, while the Hg-resistance proved to be outstanding (>0.075 mM, Table 1). This broad range of metal resistance raises the possibility that M2a might have evolved in a metal-polluted environment.

### Mobile genetic elements in M2a

#### Screening for integrons

Resistance determinants are often associated with mobile genetic elements (MGE) such as plasmids, transposons and integrons^34^. Thus the WGS of M2a was thoroughly screened for such elements. Fourteen integrase-like genes encoding putative site-specific recombinases were found in the annotated scaffolds (Suppl. data 3), but all appeared to be related to phage integrases and none to integron integrases. BLAST searches for sequences of primer pairs designed to detect genes for IntI classes 1 to 3^49^ and class 1 integron cassettes^50^ were also negative, indicating that M2a genome does not carry integrons.

#### Characterization of transposable elements

In contrast to integrons, a large number of IS elements were found in the M2a genome by BLAST searches against the IS Finder database and additional, yet unidentified elements were discovered during the thorough analysis of scaffold termini. 46 of the 277 scaffolds represented full length or partial ISs without flanking sequences, and 200 of the remaining 231 scaffolds ended in IS sequences at either (38 scaffolds, 16.5%) or both termini (162 scaffolds, 70.1%). Due to the fragmentation of the WGS, the number of IS elements could not be determined by simple counting (see Suppl. Methods). According to our estimation, 256 ISs, including 201 full length and 55 incomplete copies, occur in the chromosome and plasmids of M2a. No IS elements were identified with 100% identity to ISs available in IS Finder database. The divergence from the closest relatives in the database ranged from 99.8% DNA similarity to the level of homology undetectable by MegaBLAST comparison. In the latter cases, the new elements were classified according to the protein sequence of their putative transposase. The ca. 256 elements represent more than 55 IS species from 15 different families (Suppl. Table S2a). The exact number of IS species was hard to determine as in M2a we found many sequence variants of closely related elements that could be classified as different species or iso-elements (iso-ISs) depending on their sequence divergence. Unfortunately, there is no widely accepted cut-off value of nucleotide sequence identity for separation of IS species. IS Finder attributes new names to ISs when the similarity for protein and DNA sequences is <98% or <95%, respectively (P. Siguier, pers. comm.), and this practice was adopted in this work. Finally, 22 newly identified full length ISs have been named and submitted to IS Finder and many further IS sequences, mostly incomplete or frameshift-mutant elements, were identified as new ISs without designation (highlighted by red in Suppl. Table S2a).

#### IS families and transposons in M2a

Several IS families are represented by a single IS copy, such as families IS*21*, IS*256*, IS*481* and IS*1595*, or by only few copies, like families IS*6* and IS*NCY*. In contrast, IS*30* and IS*701* families are also represented by one IS species (isoIS*Aba125* and isoIS*Aba11*, respectively), but with more than 10 copies. The other seven families include at least three different IS species and often more than 30 copies.

The 35 IS*1*-family elements could be classified into three IS species: isoIS*Pa14* and other two types that are only 71% similar to each other and highly divergent from IS*Pa14*. Thus, the latter two were assigned as new IS*1*-family elements, IS*Alw2* and IS*Alw3*.

In M2a ca. 10 IS*3*-family ISs with more than 40 copies were found. All four subfamilies were represented and six new IS species could be identified. The most abundant ISs belonged to isoIS*Aba14* and the isoIS*Aba18/19/29/34* complex. These ISs are present in several different variants in the M2a genome and show 3–12% divergence from their closest known relatives. Out of the four fully assembled IS*51*-subfamily copies, the partial left and right end sequences found at scaffold termini could not obviously be paired due to different levels of their homology to the prototype ISs. Thus, the number of different IS species could not be exactly defined in the isoIS*Aba18/19/29/34* group (Suppl. Table S2a).

IS*4* is the next dominant family represented by 42 copies of seven IS species. 21 copies are iso-elements of IS*Aba1*, IS*Aba33* and IS*Abe18* and are almost identical to their prototype. The other copies belong to four completely new IS species (IS*Alw7*-IS*Alw10*). IS*Alw8* and IS*Alw9* copies form two slightly divergent sub-types, while IS*Alw10* copies are uniform.

The ISs with the most copies (55) in M2a genome belong to the IS*5* family, which is represented by all three subfamilies and 12 IS species. Besides the most abundant element, isoIS*Aba31*, four new members of the IS*427* subfamily were discovered. IS*Alw11* shows marginal homology to IS*Abe13* and could only be classified according to its transposase protein sequence, as well as the two different IS*Pssp5*-related elements, IS*Alw12* and IS*Alw13*. Four different ISs represent the IS*903* subfamily, three of them are closely related to IS*17*, IS*Aha2* and IS*Aba12*, respectively, while a truncated element appeared to be a new IS related to IS*Aba40*. Three further elements were classified into the IS*L2* subfamily. In addition to the slightly divergent copies of isoIS*Aba27*, two new IS species, IS*Alw14* and IS*Alw15*, distantly related to IS*Caa2* and IS*Caa3*, respectively, were identified (Suppl. Table S2a).

M2a carries 12 IS*66*-family IS copies. Except isoIS*Aba17*, isoIS*Aba25* and the newly identified IS*Alw16* and IS*Aba49*-related elements, the other copies could not confidently be classified, as their left and right parts show different levels of homology to IS*Aba25*, IS*Aba46* and IS*Aba49*. These elements differ not only from the prototype ISs but also from each other, and they represent at least five variants.

The next family, IS*630*, is represented in M2a by three species. Besides isoIS*Aba44*, two new elements were identified. IS*Alw17* is a distant relative of IS*Aba44*, while IS*Alw18* is distantly related to IS*Mae24*. Two types of IS*Alw18* occur in the chromosome: the right inverted repeat (IRR) of one copy differs at 3 positions from that of the other four identical copies. Interestingly, the single iso-element with ‘divergent’ IRR is more prevalent in other *Acinetobacter* strains, e.g. *A. lwoffii* ZS207 and *A. wuhouensis* WCHAW010062 (GenBank CP033133.1), harbouring three and 28 identical copies, respectively. Moreover, slightly different IS*Alw18* copies with the same ‘divergent’ IRR are also present in several *A. lwoffii* plasmids (pALWEK1.1, pALVED3.6).

Finally, 16 IS*982*-family elements were also found: IsoIS*Aba9* and isoIS*Aba825* copies are almost identical to the prototype elements, while isoIS*Acsp2* copies show larger divergence (87–96% similarity). Furthermore, two new family members, IS*Alw19* and IS*Alw20* were discovered. Based on their transposase protein sequence, their closest relative is IS*Neu1*, although their DNA sequences are very different.

In addition to the IS elements, three different transposons were identified in M2a. Besides the incomplete Tn*As2*-related Tn*3* family transposon, carrying the mercuric resistance (*mer*) module (Table 2), a Tn*7*-related element was found in the chromosomal scaffold sc_30. Although the termini of this element could not be exactly determined, the presence of a complete gene set (*tnsABCDE*) characteristic for Tn*7* transposons and the occurrence of close (90–95% similar) relatives of this Tn in several *Acinetobacter* strains (*A. schindleri* SGAir0122, *A. johnsonii* IC001) suggest that it is an intact transposon. In addition, a compound transposon, named as Tn*6682*, consisting of two directly oriented identical isoIS*Aba14* copies bracketing an alkyl sulfatase and a *tetR*-family regulator gene was also identified. The same 5.5 kb transposon (with 100% identity) occurs in *A. ursingii* M3 (AP018824.1), indicating a recent interspecific lateral transfer event. Further compound transposon-like structure was found in sc_211, where two inversely oriented isoIS*125* copies surround an ORF encoding a protein of unknown function. Since a similar transposon-like unit does not occur in GenBank entries, there is no indication of its transposition.

For comparison, similar analysis was carried out for *A. lwoffii* ZS207, which appeared to be the closest sequenced relative of M2a. Strain ZS207 proved to carry a similar set of ISs, but the copy number was roughly half of what we found in M2a (Suppl. Table S2b). There are 14 common IS families of the two strains, although M2a contains a new IS*1595*-family element (IS*Alw21*) that is missing from ZS207, but lacks IS*200*- and IS*L3*-family elements. Altogether, 123 copies of 51 IS species were found in the chromosome and plasmids of ZS207. 12 ISs newly identified in M2a and an incomplete copy of the new Tn*7*-like transposon are also present in ZS207. 9 further new ISs were identified, some of them appeared to be distant relatives of several new elements found in M2a. In general, the copy number of ISs is lower in ZS207 than in M2a. While the maximum copy number in ZS207 is 8 and most elements occur in less than five copies, M2a has seven ISs with 10–22 copies and 25 ISs occur in at least five copies.

A similarly high number of IS elements has been reported in the *A. baumannii* strain SDF isolated from body lice, but in contrast to the remarkable diversity of ISs of M2a, its IS population exclusively contains hundreds of IS*Aba6* and IS*Aba7* copies. This might have important role in genome reduction of their host by recombination and gene disruptions^51^.

### Plasmids of strain M2a

#### Identification of the different replicons

**Figure 2:**
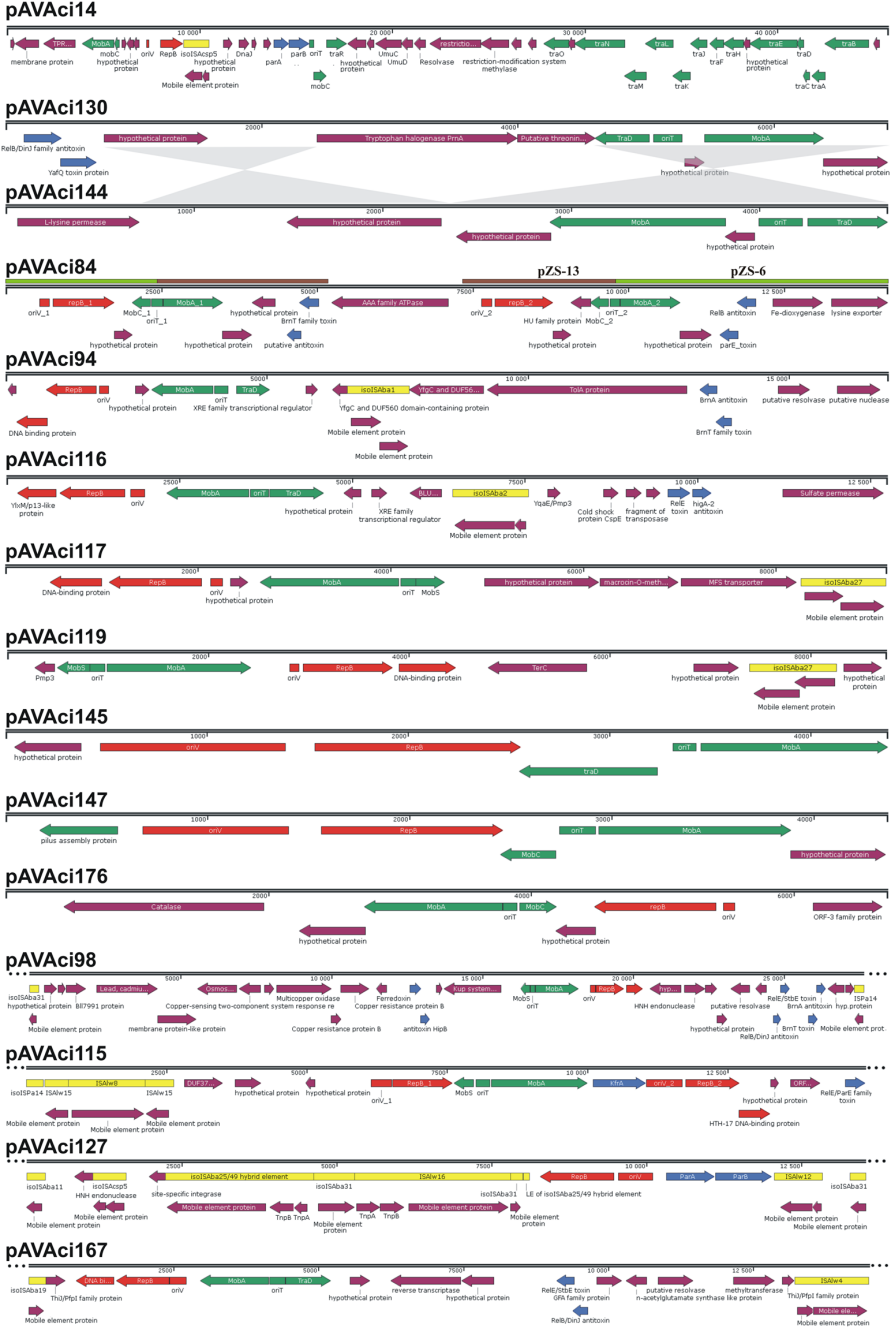
Schematic maps of plasmids identified in M2a. The colour code is: red, *rep* region (*oriV*, *repB*, DBP gene); green, mobilization; blue, TA systems; yellow, IS elements; purple, cargo genes. The related segments of pAVAci130 and pAVAci 144 are shown by grey. The regions of pAVAci84 homologous to pZS-13 and pZS-6 are indicated above the plasmid map by brown and light green bars, respectively.

Like some other *A. lwoffii* strains isolated from permafrost or arsenic-polluted environments, such as strains ED9-5a, ED23-35, ED45-23, EK30A^52^ or ZS207, M2a also proved to carry multiple plasmids (Suppl. Fig. S1). Many different plasmid sequences identified mostly in *Acinetobacter* strains were retrieved from GenBank by BLASTn search with the 57 plasmid-related scaffolds found in the WGS (Supplementary data 1). By screening the WGS for plasmid-related genes, such as genes for replication initiation (*rep*) and conjugal transfer (*tra, mob*), 15 plasmids could be identified (Table 3, Fig. 2), which appears to be exceptional compared to the mentioned strains that have 8–12 plasmids^52^, (ZS207: CP019144 to CP019152).

**Table 3:** List of plasmids identified in M2a and their closest relatives found in GenBank.

Plasmid name	Scaffold	Acc No.	Length (bp)	Mean G + C (%)	Plasmid related genes	Best BLASTn hit(s)	Acc No.	Host strain	Coverage/Identity (%)
pAVAci14	sc_14	**MK978162**	45,741	35	*repB*, *mobAC*, *parAB*, *traRONMLKJFHEDCAB*	pHHV35	FJ012882.1	Uncultured	80/99
pAVAci84	sc_84	**MK944320**	14,155	35	2 *repB*, 2 *mobAC*, 2 TA,	pZS-6 pZS13	CP019146.1 CP019151.1	*A. lwoffii* ZS207	99/99 72/99
pAVAci94	sc_94-223-279-187- 268-232	**MK978163**	16,889	38	*repB*, *mobA*, *traD*, TA	pO237-4 pALWED3.2	MK431775.1 CP032287.1	*A. baumannii* 11A16CRGN004 *A. lwoffii* ED9-5a	25/92 21/92
pAVAci116	sc_116- 188-280- 190	**MK978161**	12,740	36	*repB*, *mobA*, *traD*, TA	pMS32-3 pOXA58-AP_882	KJ616406.1 CP014479.1	*A. pittii* MS32 *A. pittii* AP_882	17/94 49/94
pAVAci117	sc_117	**MK978159**	9,146	32	*repB*, *mobAS*	pAb825_36	MG100202.1	*A. baumannii*	35/93
pAVAci119	sc_119	**MK978160**	8,751	35	*repB*, *mobAS*	pM131-5	JX101644.1	*A*. sp. M131	41/90
pAVAci130	sc_130	**MK944319**	6,886	35	*mobA*, *traD, TA*	pZS-7/ pALWEK1.6	CP019147.1 CP032108.1	*A. lwoffii* ZS207 *A. lwoffii* EK30A	100/99 99
pAVAci144	sc_144	**MK944317**	4,677	37	*mobA*, *traD*	pZS-8	CP019148.1	*A. lwoffii* ZS207	100/97
pAVAci145	sc_145	**MK944318**	4,381	40	*repB*, *mobA*	pRGRH0231	LN852904.1	Uncultured	100/94
pAVAci147	sc_147	**MK944321**	4,364	37	*repB*, *mobAC, pilE*	pAba11510a	CP018862.2	*A. baumannii* 11510	71/95
pAVAci176	sc_176-150	**MK944322**	6711	39	*repB*, *mobAC*	pALWEK1.4	CP032107.1	*A. lwoffii* EK30A	77/95
pAVAci98^*a*^	sc_93-98- 177	**MK993303**	>27,622	38	*repB*, *mobA*, 3 TA	pALWED1.4 pALWEK1.1	CP032113.1 CP032102.1	*A. lwoffii* ED23-35 *A. lwoffii* EK30A	28/96 55/95
pAVAci115^*a*^	sc_219-191-168-115-278-239	**MK993300**	>14,954	38	2 *repB*, *mobAS, kfrA*, TA	pRGFK1137 pIC001C	LN853717.1 CP022301.1	Uncultured *A. johnsonii* IC001	23/99 60/98
pAVAci127^*a*^	sc_127-213-165-175	**MK993301**	>13,529	41	*repB*, *parAB*	pALWED2.1 pmZS	KX426229.1 CP019144.1	*A. lwoffii* ED45-23 *A. lwoffii* ZS207	99/98
pAVAci167^*a*^	sc_167-103	**MK993302**	>14,221	36	*repB*, *mobA*, *traD*, TA	pB8300 pZS-20	CP021348.1 CP019152.1	*A. baumannii* B8300 *A*. *lwoffii* ZS207	29/83 39/95

^*a*^Where circularization of the plasmid sequence could not be accomplished, the data refer only to the assembled contigs.

Eight plasmid sequences (pAVAci14, pAVAci84, pAVAci117, pAVAci119, pAVAci130, pAVAci144, pAVAci145 and pAVAci147) were assembled into single scaffolds by the A5-assembly. Their sequence could be circularized based on their overlapping end sequences and sealing PCRs carried out with appropriately designed primers facing outward of the ends of the respective scaffold. Further three plasmid sequences (pAVAci94, pAVAci116 and pAVAci176) could be completed by manual assembly of scaffolds based on their overlapping end sequences. In these cases PCR verification of the assembly and, if required, sequencing of the sealing PCR fragment were also accomplished. To confirm that these sequences are circular extrachromosomal elements, they were cloned (except the 46 kb pAVAci14) in an R6K-based *E. coli* plasmid using unique restriction sites found in the plasmid sequences (see Methods). These clones, maintained in TG2 λ*pir* strain, were also used to test whether the plasmids are able to replicate in *E. coli*. The cloned pAVAci plasmids were introduced into TG1 cells, which do not support the maintenance of the R6K-based replicon of the cloning vector. None of them resulted in colonies indicating that these plasmids, similar to most *Acinetobacter* plasmids, are unable to replicate in *E. coli*.

Four additional plasmids (pAVAci98, pAVAci115, pAVAci127 and pAVAci167) were identified based on their *rep* and other genes characteristic for plasmids (*par*, *mob*, toxin-antitoxin (TA) module)^53^. These plasmid sequences could not be completed even by manual assembly due to the high number of scaffolds ended with similar IS elements. The replicon regions of pAVAci98 and pAVAci127 are similar to large plasmids like pALWEK1.1, pmZS and pALWED2.1, which all carry numerous IS elements as well. However, many scaffolds represent as yet unknown sequences, which prevented the full assembly of the sequences using the published relatives as reference sequences.

#### General features of plasmids in M2a

Despite the large diversity of plasmids in M2a, some common features could be seen in their replication and mobilization regions (Fig. 2). All but two plasmids have *repB* gene coding for a Rep-3 superfamily replication initiation protein. In most cases, *repB* is followed by a putative DNA-binding protein (DBP) gene as was found in many other Rep-3 superfamily replicons^37^. The noncoding upstream region of *repB* always contains four to nine directly oriented imperfect or perfect repeats of about 20-bp motif. This part of the plasmids possibly functions as the iteron region of the replication origin (*oriV*). The iterons are often accompanied by shorter direct or inverted repeats (DR or IR, respectively) probably belonging to the entire functional *oriV*. The two exceptions are pAVAci130 and pAVAci144 that have no *repB* gene and where an iteron region could not be found.

**Figure 3:**
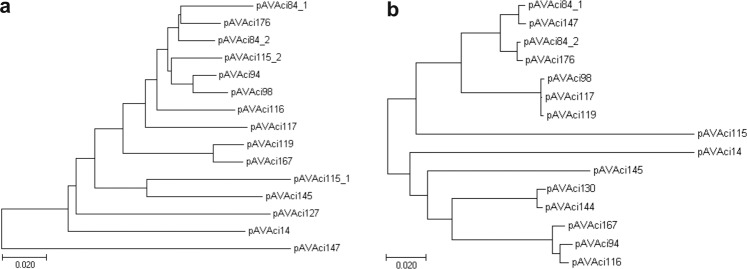
Phylogenetic relationship of the predicted RepB and MobA proteins found in M2a. Trees were generated using the neighbour-joining method. Bar, 0.02 changes per amino acid position. (**a**) Tree for RepB proteins. (**b**) Tree for MobA proteins.

The other generally occurring plasmid-related function was the mobilization genes (*mob*). Although only pAVAci14 appears to have a complete gene set for conjugal transfer, all but one other of the plasmids carry a *mob* region containing plasmid mobilization genes like *mobA/mobL* and *mobC*, *mobS* or *traD*. The common pattern of *mob* regions are the divergently oriented *mobA/mobL*-family nickase/relaxase gene and a short *mobS-*, *mobC-* or *traD-*like gene, which are separated by about 200–300 bp non-coding sequence. The localization between divergent *mob* genes and the presence of an array of IR motifs suggest that these non-coding regions contain the origin of transfer (*oriT*)^54,55^. Since these plasmids have no other transfer related genes such as genes for Type IV pilus components and assembly factors or coupling protein, it is more possible that they can be trans-mobilized by other conjugative plasmids rather then they are self-transmissible. Although, the occurrence of similar *mob* regions in Rep-3 superfamily plasmids is not extraordinary, their horizontal transfer has yet to be demonstrated^37^. The phylogenetic trees generated for the RepB and the MobA protein sequences (Fig. 3) differ significantly, which indicates that the replicons and the mobilization regions evolved mostly independently of each other and intensive reshuffling might occur between the different plasmids by recombination and transposition.

#### Characterization of the M2a-derived plasmids

The only exception to the pattern described above for *mob* regions can be seen in pAVAci14, where the putative *oriT*-like region was found between the directly oriented *parA* and *mobC* genes (Fig. 2). The closest relative of the 45.7 kb pAVAci14 is pHHV35 (Table 3) belonging to the “Low-GC” group of plasmids^56^, where *oriT* have been localized at the same position as predicted in pAVAci14. Besides the replication (*repB*, *oriV*), maintenance (*parAB*,) and transfer functions, pAVAci14 contains relatively few accessory genes (Type III restriction/modification system, *umuCD*-like repair genes, *dnaJ*, a Ser-recombinase family resolvase, an MFS-1 family membrane protein, a TPR-repeat-containing protein, several hypothetical genes and an IS*Acsp5* copy). Interesting dissimilarity to pHHV35 backbone is that pAVAci14 contains additional *mobC* and *mobA/mobL* mobilization genes encoded by partially overlapping ORFs in a single operon. These genes along with seven other ORFs are inserted amongst *traB* and *oriV* interrupting the conserved backbone (Fig. 2). Although pAVAci14 appears to have no resistance determinants, the group of conjugative Low GC plasmids have been suggested to be important factors in environmental spread and interspecies transfer of antibiotic resistance between bacterial communities of manure and soil^56–58^.

Plasmids pAVAci130 and pAVAci144 devoid of *rep* genes are highly homologous to plasmids pALWEK1.6/pZS-7 and pZS-8 (Table 3). pAVAci130 contains only one or two base substitutions compared to the published pALWEK1.6 and pZS-7 sequences, respectively, while pAVAci144 differs from pZS-8 at 134 positions (2.8%) and lacks the IS*5* family element inserted in pZS-8 near *mobA/mobL*. The two plasmids are related to each other as their mobilization regions and a 1.6 kb other segments with a hypothetical gene and a 0.9 kb non-coding sequence, which possibly includes the replication origin, show 88% and 84% similarity, respectively (Fig. 2). A ca. 670 bp tract of the non-coding regions shows 77% homology to the upstream region of *orf2* of pRAY, a representative of plasmids also lacking *rep* gene and widely distributed in *Acinetobacter*^59^. The almost identical sequence of the three plasmids, pAVAci130, pZS-7 and pALWEK1.6, which were found in different strains of *A. lwoffii* isolated at different time and locations, suggests recent horizontal transmission of this plasmid, which indirectly supports the assumption of its ability for trans-mobilization. Interestingly, pALWEK1.6 derives from *A. lwoffii* strain EK30A that was isolated from permafrost of 1.6–1.8 million-year-old Pleistocene sediment^52^, which might suggest that the evolution of this plasmid is exceptionally slow (1 base substitution/1.6 M year) or the strain EK30A was a recent environmental contamination in the permafrost sample.

Plamsid pAVAci84 appears to be the fusion product of two plasmids as it carries two complete *rep* and *mob* regions and two TA systems. The RepB proteins are closely related (Fig. 3a) and the putative *oriV*s are also similar. Both carry an array of a 9-bp AT-rich direct repeats, however, the following array of four 22-bp repeats (the iterons) are different. Both *mob* regions consist of divergent *mobC* and *mobA* genes and an *oriT*-like sequence between them. MobA proteins are also closely related, however, similarly to the RepB-s, they are not the closest relatives in M2a (Fig. 3b). The presumed fusion of the two plasmids resulting in pAVAci84 seemingly occurred between the putative *oriT* regions of the parental plasmids. One component of pAVAci84 (1–2409 and 9769–14155 bp) is almost identical to pZS-6. The only differences are 3 SNPs and the duplication of a 206-bp tract downstream of the Fe-dioxigenase gene. The other component, however, is more closely related to pZS-13, from which it differs only in 6 SNPs and in the presence of an AAA-family ATPase gene inserted between the *brnT*-family toxin gene and the *rep* region (Fig. 2). The sequence of pZS-6 and pZS-13 contains several regions showing 76–82% similarity, but one of the longest identical sequences occurs in their putative *oriT*s, which might explain that the fusion took place at this region. Although the formation of pAVAci84 can be explained by homology-dependent recombination, the involvement of the putative relaxases in this process cannot be excluded.

The 17-kb pAVAci94 appears to be a new plasmid species. Its *rep* region, consisting of *oriV*, *repB* and a putative DBP gene, and the *brnA*-*brnT* TA system show 92% and 88% homology to the respective regions of the *A. baumannii* plasmid pO237-4. In contrast, the *mob* region is 88% similar to that of pALWED3.2 (Table 3). Additional parts of pAVAci94 (from *yfgC* to a group I intron nuclease-domain-containing protein gene) has no homologs among DNA entries of GenBank.

The 13-kb pAVAci116 also seems to be a new plasmid species with mosaic structure. Its *rep* region (*oriV, repB* and a DBP gene *ylxM*) is similar to that of pMS32-3, however, the *mob* region (*mobA-oriT-traD*), the TA system and a sulphate permease gene are related to those of pOXA58-AP_882 (Table 3).

The ca. 9-kb plasmids pAVAci117 and pAVAci119 have closely related *mob* regions, while their *rep* regions are more dissimilar (Fig. 3). The *rep* region of pAVAci117 is only partially homologous to several *A. baumannnii* plasmids (e.g. pAb825_36 or p597A-14.8), while that of pAVAci119 is 82–85% homologous to other *A. baumannnii* plasmids such as p2ABSDF or pEH_gr3. Almost half of pAVAci117, coding for three cargo genes, has no homologs in the database.

The two smallest plasmids, pAVAci145 and pAVAci147 do not carry accessory modules besides the basic replicon and the *mob* region (Fig. 2). The putative DBP gene generally located downstream of *repB* is missing from both plasmids and their RepBs, and the *mob* regions are quite different (Fig. 3). The *mob* region of pAVAci145 consists of *mobC-oriT-mobA*, while the other plasmid has a *traD*-like gene instead of *mobC* and their MobA proteins are also located on different branches of the phylogenetic tree (Fig. 3b).

The last small plasmid, pAVAci176 also lacks a DBP gene, however the ORF downstream of *repB* might have similar function. The *mob* region corresponds with the *mobC-oriT-mobA* pattern and the cargo module, except the ORF-3-like protein gene, is related to that of pALWEK1.4.

Among the remaining plasmid-related scaffolds, representing 276.8 kb sequence, four were found that carried similar *rep* and *mob* regions identified in the fully assembled plasmids. The scaffold sc_98 contained a complete Rep-3-type replicon (*oriV-repB*-DBP gene) and a *mob* region (*mobS-oriT-mobA*). Two large scaffolds could unambiguously be joined to sc_98 and the resulting sequence carried three TA-systems and a gene set for resistance to heavy metals (Fig. 2). A BLAST search with this segment as query indicated that this plasmid, designated as pAVAci98, is most closely related to the large plasmid pALWEK1.1 which also carries metal resistance modules.

The next plasmid-derived scaffold was sc_115, which carried two different replicons and a *mobS-oriT-mobA*-type *mob* region. The two replicons are not closely related as one of the RepB proteins is closer to RepB of pAVAci145, while the other forms a distant branch with those of pAVAci84, pAVAci94, pAVAci98 and pAVAci176. The *rep_1* region consists of *repB* and *oriV* assembled from five 11-bp repeats followed by nine 23-bp iteron repeats, while *rep_2* includes an additional HTH-17 type DBP gene and the *oriV* contains four 22-bp iteron repeat preceded by a complex array of IRs. Accordingly, the closest relatives of *rep_1* + *mob* and *rep_2* regions were found in different plasmids, i.e. pRGFK1137 and pIC001C, respectively (Table 3). The backbone of this plasmid, named as pAVAci115, also carries a TA-system and a *kfrA*-like gene, which may participate in plasmid maintenance. Sc_115 could be joined to several short scaffolds, which added some cargo genes and a complex IS-in-IS segment to the backbone (Fig. 2), however it could not be completed.

pAVAci127 was the only plasmid that lacks a *mob* region. Its basic replicon (*repB* and *oriV*, no DBP) and the plasmid partitioning genes *parAB* are bracketed by tracts of IS elements (Fig. 2). The *rep-par* segment is almost identical to the homologous parts of the 190–200 kb plasmids pALWED2.1 and pmZS. These plasmids contain many metal-resistance determinants and, similarly to pAVAci127, seem to be not mobilizable.

The last replicon identified in M2a was pAVAci167. Its *rep* region found in sc_167 contains a DBP gene, *repB* and the *oriV*. The *mob* region shows the *traD-oriT-mobA* pattern. The closest relatives of pAVAci167 (based on the *rep* and *mob* regions) are 13–25 kb plasmids identified in *A. baumannii* and *A. pittii* strains (i.e. pB8300), however pAVAci176 carries a ca. 5 kb region containing a TA-system, a resolvase and a methyltransferase gene, which is 95% similar to a segment of pZS-20 (Table 3).

Since pAVAci115 and pAVAci167 are related to smaller (7–30 kb) plasmids, while pAVAci98 and pAVAci127 show similarity to large (~200 kb) plasmids like pALWEK1.1, pmZS or pALWED2.1, it is presumable that most of our unassembled plasmid scaffolds (Suppl. data 1) belong to pAVAci98 and pAVAci127. These parts of plasmids carry lots of IS elements, code for metal resistance efflux systems and many metabolic (oxidases, reductases, membrane transporters) and unknown functions, which may contribute to the adaptability of the host organism.

## Conclusions

*A. lwoffii* strain M2a that was isolated from a Transylvanian honey sample derived from a nearly natural environment proved to contain 15 different plasmids, more than 250 IS elements of 15 IS-families and some unit and compound transposons. Besides several antibiotic efflux systems and an OXA-134 family β-lactamase gene, the strain carries numerous chromosomal and plasmid-borne heavy-metal resistance determinants similarly to *A. lwoffi* strains isolated from metal-polluted environments or permafrost^52^. One out of its 15 plasmids, the “Low GC” family plasmid pAVAci14, has an apparently complete conjugative system, while the others, except pAVAci127, have a *mob*-region showing common pattern. All *mob* regions consist of a divergently oriented *mobA/mobL*-family relaxase gene and a *mobS-*, *mobC-* or *traD-*like gene separated by a putative *oriT* of about 200–300 bp with several IR motifs. The frequent occurrence of such *mob* regions suggests that these plasmids are *trans* mobilizable, perhaps by pAVAci14. Plasmid pAVAci130 of M2a are almost identical to plasmids of *A. lwoffi* strains isolated recently from metal-polluted environments and from 1.6 million-year-old permafrost sediment^52^. The first case may indicate a recent lateral transfer event, however the second is hard to explain without supposing that pAVAci130/pALWEK1.6 evolved extremely slowly (1 base substitution per 1.6 M years) or that the source strain EK30A was a recent contaminant in the permafrost sample. Regarding *A. lwoffii* M2a, it was probably a bee-delivered contamination^27^ in the honey sample it was isolated from. Although M2a does not show extensive antibiotic resistance, its several plasmids are related to factors of environmental spread of AR between bacterial communities^56–58^. The high number of its presumably transferable plasmids and the outstanding number and diversity of IS elements that may be involved in reshuffling the chromosomal and plasmid-borne gene content, may indicate the potential of such strains to rapidly become a multiresistant pathogen^60–62^, which should not be overlooked.

## Methods

### Isolation of bacteria from honey samples

The project initially aimed to isolate lactobacilli and other bacteria from honey produced in nearly natural environment of Transylvanian meadow near Székelykeresztúr (Cristuru Secuiesc, Romania). The honey samples were collected in 2014 and stored at room temperature until the analysis (isolation of bacteria occurred within two months after sample collection). Approximately 2 g of honey samples were suspended in 15 ml peptone water (0.1% w/v, 0.5% NaCl, pH 7.2), then were centrifuged (10 min, 25 °C, 3000 × g) and the pellet was resuspended in 500 µl peptone water. Twenty-five μl suspension was plated on MRS agar^63^ with or without 0.8% CaCO_3_ and incubated for 48 h under CO_2_-enriched condition (in the presence of 5% CO_2_) at 35 °C. Strain M2a was isolated from MRS agar + CaCO_3_ plate. The original colony was streaked twice on LB agar plates and grown under aerobic condition, which appeared more convenient to maintain the strain.

### Microbial techniques and biochemical tests

M2a and *E. coli* strains were grown at 30 °C or 37 °C, respectively, in Luria-Bertani (LB) broth or agar plates and were maintained at −70 °C in LB broth containing 30% glycerol. The MICs for antibiotics and heavy metals for M2a (Table 1) were determined by the agar dilution method^64^ with minor modifications. Bacteria were grown overnight at 30 °C in LB broth and then the culture was serially diluted 10-fold to 10^7^ × with 0.9% NaCl solution. Five μl of the bacterial suspensions (cc to 10^7^ × dilutions of the 2.5 × 10^8^ CFU/ml culture) was dropped onto LB plates containing different concentrations of the examined antibiotics or heavy metal salts. The used concentrations of antibiotics (μg/ml) were as follows: ampicillin: 50, 75, 100, 125, 150, 175, 200; chloramphenicol: 5, 10, 15; ciprofloxacin: 0.25, 0.5, 075, 1.0, 3.0, 6.0; erythromycin: 25, 50, 75, 100, 125, 150; florphenicol: 10, 15, 20, 25; gentamycin: 2, 4; kanamycin: 10, 15, 20; nalidixic acid: 5, 10, 15; neomycin: 10, 20, 30, 40; rifampicin: 25, 50, 75, 100; spectinomycin: 20, 50, 60, 70, 100, 125, 150, 300; streptomycin: 10, 25, 50; tetracycline: 2, 4; zeocin: 25, 50, 75, 100; and of metal ions (μM): HgCl_2_ (Hg^2+^): 15, 30, 45, 60, 75; CdCl_2_ × H_2_O (Cd^2+^): 10, 25, 50, 100, 200, 400, 600, 800, 1000; CoCl_2_ × 6H_2_O (Co^2+^): 10, 25, 50, 100, 200, 400, 600, 800, 1000; CuSO_4_ × 5H_2_O (Cu^2+^): 900, 1800, 2700, 3600, 4500; ZnSO_4_ × 7H_2_O (Zn^2+^): 200, 400, 800, 1600, 3200. Concentration of viable cells (CFU/ml) was determined on LB plates without antibiotics or heavy metal salts. The plates were incubated at 30 °C for 24 h and visually evaluated. MIC value was determined on the 100 × dilution drops (ca. 1.25 × 10^4^ cells/drop).

Biochemical tests e.g. methyl red, Voges-Proskauer, indole, citrate utilization, catalase production, urease, and oxidase tests were performed as described previously^65^. Glucose, lactose and sucrose fermentation, and gas and H_2_S production was examined on Triple Sugar Iron agar (Biolab Inc., Budapest, Hungary). Growth was tested at 30/37/44 °C for 1 days on Luria–Bertani (LB) and Eosin Methylene Blue agar (Biolab Inc., Budapest, Hungary).

### DNA purification and sequencing

Total DNA for WGS was purified from M2a using Qiagen Blood & Cell Culture Kit with Genomic-tip 20/G (Qiagen, Hilden, Germany) according to the manufacturer’s instructions. DNA quality and quantity were tested on Ethidium-bromide-stained agarose gel (1% agarose, 1% TBE buffer) and NanoDrop ND 1000 spectrophotometer (NanoDrop Technologies, Wilmington, DE, USA). The 600-630-bp fragment library was prepared by UD GenoMED (Debrecen, Hungary) and 2 × 300-bp paired-end genome sequencing was performed by University of Szeged, Department of Biochemistry and Molecular Biology (Szeged, Hungary) as a custom service using Illumina’s MiSeq platform.

For cloning and PCRs plasmid DNA of M2a was extracted from 100 ml culture using the QIAGEN Plasmid Midi Kit (QIAGEN, Hilden, Germany) according to the manufacturer’s instructions. Cloning procedures and transformation of CaCl_2_ competent *E. coli* cells were carried out according to^66^. M2a-derived plasmids were cloned in the *pir*-dependent vector pSG76C^67^ and maintained in *E. coli* strain TG2 λ*pir*, a derivative of TG2^66^ obtained by lysogenization with λ*pir* phage isolated from S17-1 λ*pir* strain^68^. Colony PCRs were performed using Dream Taq polymerase (Thermo Fisher Scientific) as described previously^69^. Oligonucleotide primers used in this work are listed in Suppl. Table S3. Sanger sequencing was carried out on ABI Prism 3100 (Perkin Elmer) by BIOMI Ltd. (Gödöllő, Hungary). The bacterial isolates were first classified by sequencing of their 16S rDNA segment amplified in colony PCR using primers 27for and 1492rev^70^.Thermal cycling was as follows: initial denaturation at 96 °C for 5 min, followed by 35 cycles of 95 °C for 30 s, 55 °C for 30 s and 72 °C for 90 s and a final extension at 72 °C for 7 min.

### Bioinformatics

The Illumina MiSeq reads were de novo assembled into scaffolds using A5-miseq pipeline^71^. The scaffolds were annotated using the RAST server^72^. The Whole Genome Shotgun project has been deposited at DDBJ/ENA/GenBank under the accession VCND00000000. The version described in this paper is version VCND01000000. The scaffolds of WGS available in GenBank was re-annotated by the NCBI’s annotation server. The completed plasmid sequences identified in M2a have also been deposited at GenBank under the accession numbers listed in Table 3.

All homology searches were carried out using BLAST^73^ in the NCBI database (http://www.ncbi.nlm.nih.gov/). Alignment of M2a scaffolds to the reference *A. lwoffii* strain ZS207 chromosome was carried out using Mauve^74^. For the phylogenetic reconstructions ClustalW and Neighbour-joining tree algorithm of MEGA7 was used with the default settings^75^.

AR determinants in WGS of M2a strain were searched using The Comprehensive Antibiotic Resistance Database (CARD)^76^, MEGARes^77^ and ARG-ANNOT^78^. INTEGRALL database^79^ and IS Finder^80^ were applied for searches of integrons and IS elements, respectively.

### Completion of plasmid sequences and cloning of plasmids

For sealing the sequences of the putative plasmid scaffolds primers facing outward of the ends of scaffold sequences were designed (Suppl. Table S3) and PCRs were carried out using the respective primer pairs and plasmid DNA template isolated from M2a. PCR cycling was: initial denaturation at 94 °C for 2 min, followed by 35 cycles of 94 °C for 20 s, 55 °C for 30 s and 72 °C for 1 min and a final extension at 72 °C for 5 min. The PCR fragments obtained were sequenced on ABI Prism 3100 Genetic Analyzer (Perkin Elmer). The plasmid sequences assembled from scaffolds sc_93-98-170, sc_127-213-165, sc_167-103, and sc_219-191-168-115-278-239 could not be circularized even by long PCRs carried out using the appropriate primer pairs as follows: initial denaturation at 94 °C for 1.5 min followed by 10 cycles of 94 °C for 20 sec, 55 °C for 30 sec and 68 °C for 7 min, and 20 cycles of 94 °C for 20 sec, 55 °C for 30 sec and 68 °C for 7 min + 5 sec/cycle and a final extension at 68 °C for 10 min.

For cloning the circular plasmids into the R6K-based *pir*-dependent *E. coli* vector pSG76-C, a unique restriction site located out of the potential replication regions of each plasmid was applied. The plasmids pAVAci84 and pAVAci176 were linearized with *Pst*I, pAVAci94 and pAVAci117 with *Sal*I, pAVAci119 with *Xma*I and pAVAci145 with *Eco*RI and all were ligated into the corresponding sites of pSG76-C. pAVAci116 and pAVAci147 were cleaved with *Nru*I and ligated into the *Sma*I site. pAVAci130 and pAVAci144 were cleaved with *Xho*I and *Mfe*I, respectively, and ligated into the *Sal*I and the *Eco*RI sites. The resulting pSG76-C derivative plasmids were maintained in *E.coli* TG2 λ*pir* cells. The ability of cloned M2a-derived plasmids for autonomous replication in *E. coli* was tested by transformation into *E. coli* strain TG1^66^. For selection of transformant *E. coli* strains 20 μg/ml chloramphenicol was used in the culture media.

## Supplementary information

Supplementry Information

Supplementry Table S2

## Data Availability

All data generated or analysed during this study are included in this published article (and its Supplementary Information files) or available in public databases.
