# Supplementary material for: Abundance of mobile genetic elements in an Acinetobacter lwoffii strain isolated from Transylvanian honey sample
Source: Sci Rep. 2020 Feb 19;10:2969. doi: 10.1038/s41598-020-59938-9 (PMC7031236; doi:10.1038/s41598-020-59938-9)
Supplement: Supplementary file 1 — Supplementry Information [file 41598_2020_59938_MOESM1_ESM.pdf]

A. VERESS, T. NAGY, T. WILK, J. KÖMÜVES, F. OLASZ, J. KISS: Abundance of mobile genetic elements in an *Acinetobacter lwoffii* strain isolated from Transylvanian honey sample

Supplementary Figure S1-S3

Supplementary Table S1, S3

(Supplementary Table S2 is a separate Excel file)

Supplementary Data 1, 2, 3

Supplementary Methods

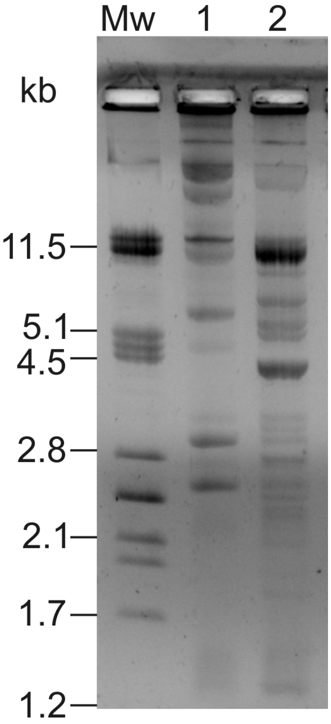


**Supplementary Fig. S1.** Plasmid DNA isolated from strain M2a. Lane 1, undigested plasmid DNA; lane 2, plasmid DNA digested with *Eco*RI; Mw, λ DNA digested with *Pst*I. (The uncropped original gel image can be seen below).

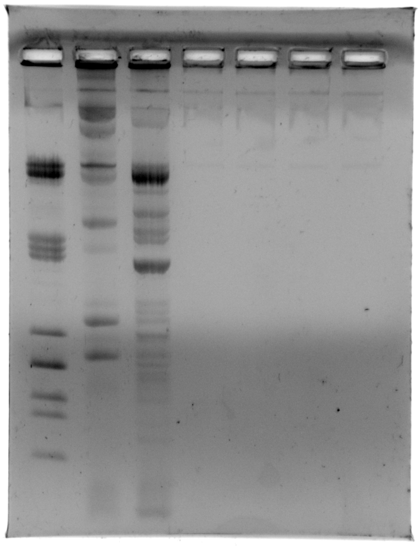

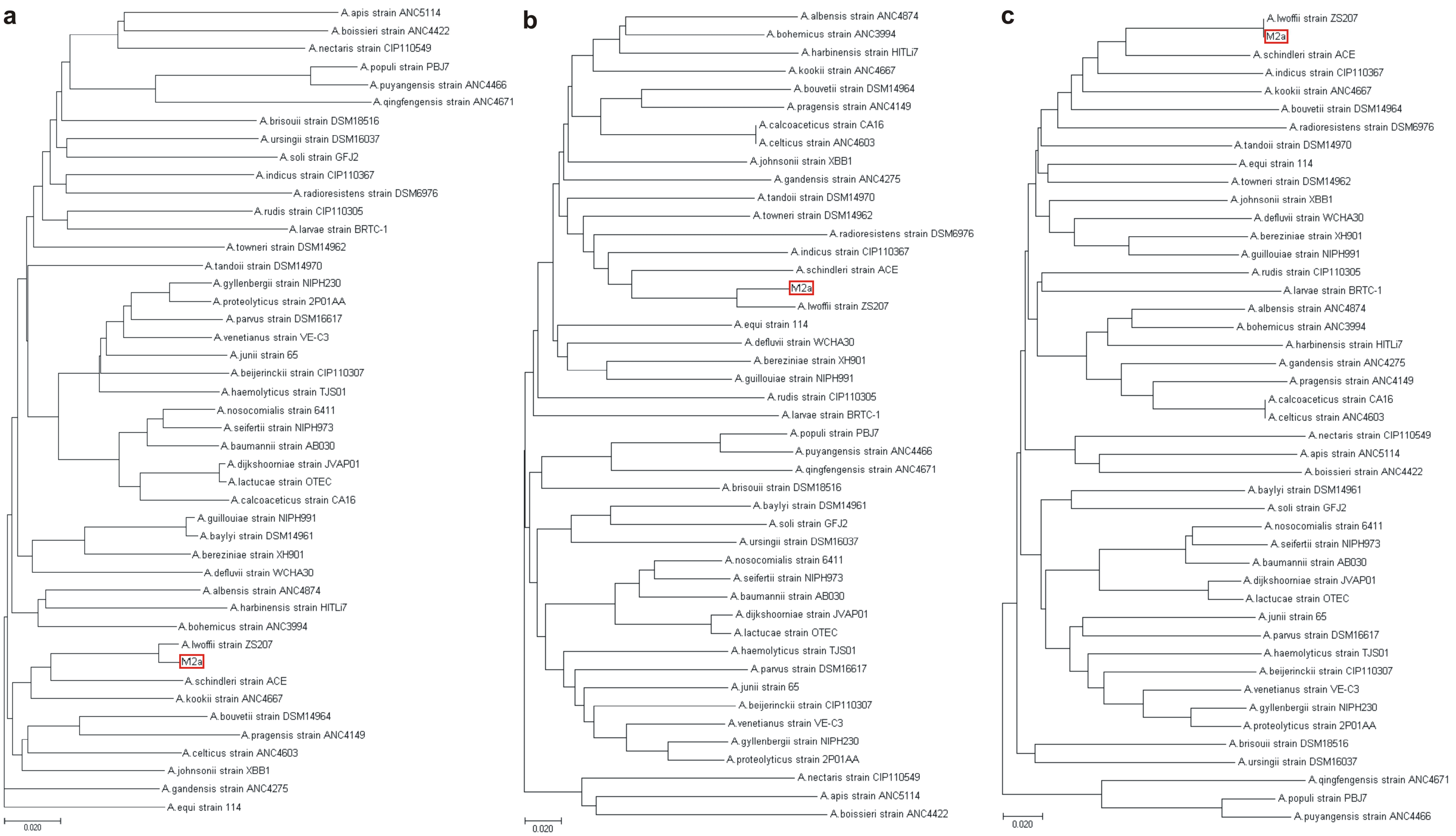


**Supplementary Fig. S2.** Phylogenetic trees generated using the neighbour-joining method for *rpoB* (**a**), *gyrB* (**b**) and *recA* (**c**) genes of strain M2a and 44 representatives of *Acinetobacter* species. Bars, 0.02 changes per nucleotide position.

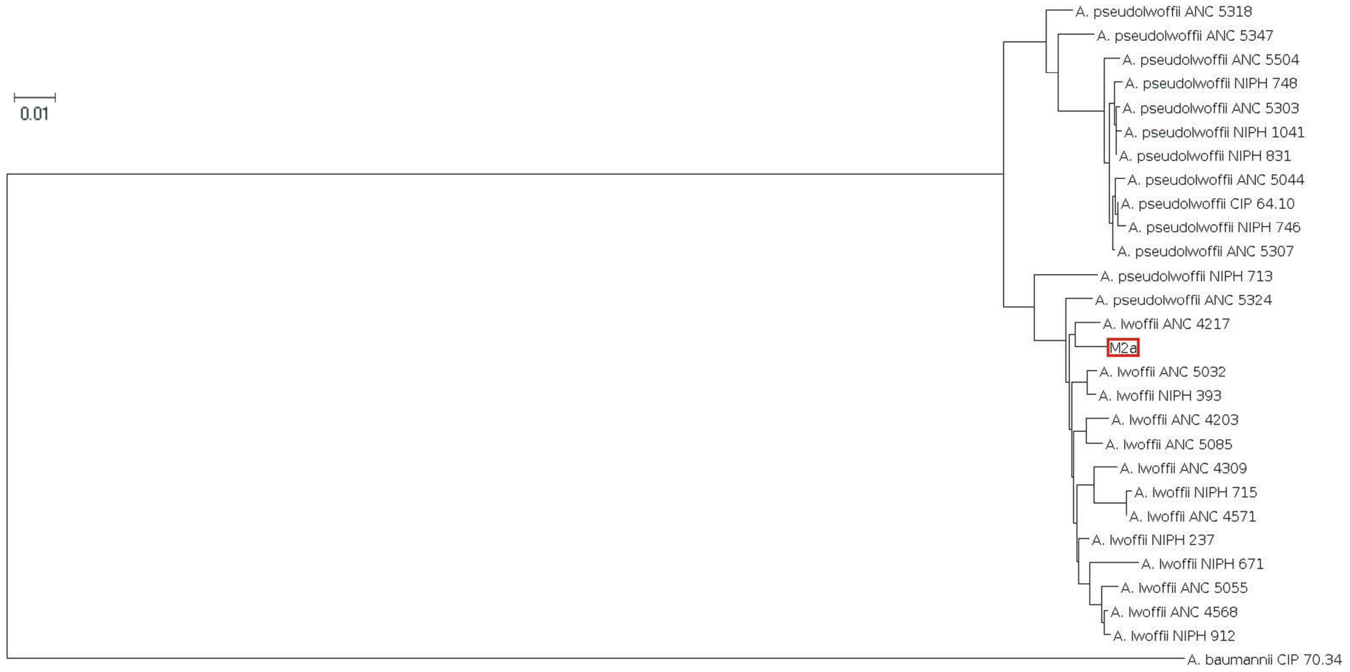


**Supplementary Fig. S3.** Phylogenetic tree generated using the neighbour-joining method for the 861 bp fragment of *rpoB* gene [1] of strain M2a and 13 representatives of *A. lwoffii* and *A. pseudolwoffii* species [1]. Bar, 0.01 changes per nucleotide position.

Reference

1. Nemec, A. *et al.* Revising the taxonomy of the *Acinetobacter lwoffii* group: The description of *Acinetobacter pseudolwoffii* sp. nov. and emended description of *Acinetobacter lwoffii*. *Syst. Appl. Microbiol.* **42**, 159–167 (2019).

**Supplementary Table S1**

**a. Species/strains from nine genera of *Moraxellaceae***

| Species/strains from nine genera of *Moraxellaceae* | Accession No. |
|---|---|
| *Acinetobacter baumannii* AB030 | CP009257 |
| *Acinetobacter nectaris* CIP 110549 | KI530712 |
| *Acinetobacter lwoffii* ZS207 | CP019143 |
| *Alkanindiges hongkongensis* HKU9 | AF543466 |
| *Cavicella subterranea* W2.09-231 | JX458449 |
| *Faucicola mancuniensis* GVCNT2 | KC688888 |
| *Fluviicoccus keumensis* | KF891388 |
| *Moraxella lacunata* | AF005160 |
| *Paraperlucidibaca baekdonensis* RL-2 | GU731671 |
| *Perlucidibaca* sp. W2.10-39 | JX458465 |
| *Psychrobacter aestuarii* NIOMR13 | KY673001 |

**b. *Acinetobacter* species/strains**

| | |
|---|---|
| *A. albensis* ANC 4874$^{T}$ | NZ_FMBK00000000 |
| *A. apis* ANC 5114$^{T}$ | NZ_FZLN00000000 |
| *A. baumannii* AB030 | NZ_CP009257 |
| *A. baylyi* DSM 1496 | JHZI01000000 |
| *A. beijerinckii* CIP 110307$^{T}$ | APQL01000000 |
| *A. bereziniae* XH901 | NZ_CP018259 |
| *A. bohemicus* ANC 3994$^{T}$ | APOH01000000 |
| *A. boissieri* ANC 4422$^{T}$ | NZ_FMYL00000000 |
| *A. bouvetii* DSM 14964$^{T}$ | APQD01000000 |
| *A. brisouii* DSM 18516 | BBTI01000000 |
| *A. calcoaceticus* CA16 | NZ_CP020000 |
| *A. celticus* ANC 4603$^{T}$ | NZ_MBDL00000000 |
| *A. defluvii* WCHA30$^{T}$ | NZ_CP029397 |
| *A. dijkshoorniae* JVAP01$^{T}$ | NZ_KM923969 |
| *A. equi* 114$^{T}$ | NZ_CP012808 |
| *A. gandensis* ANC 4275 $^{T}$ | NZ_LZDS00000000 |
| *A. guillouiae* NIPH 991 | APPJ01000000 |
| *A. gyllenbergii* NIPH 230 | AYEQ01000000 |
| *A. haemolyticus* TJS01 | NZ_CP018871 |
| *A. harbinensis* HITLi 7$^{T}$ | NZ_JXBK00000000 |
| *A. indicus* CIP 110367 | AYET01000000 |
| *A. johnsonii* XBB1 | CP010350 |
| *A. junii* 65 | CP019041 |
| *A. kookii* ANC 4667$^{T}$ | NZ_FMYO00000000 |
| *A. lactucae* OTEC-02 | NZ_CP020015 |
| *A. larvae* BRTC-1$^{T}$ | NZ_CP016895 |
| *A. lwoffii* ZS207 | CP019143 |
| *A. nectaris* CIP 110549$^{T}$ | AYER01000000 |
| *A. nosocomialis* 6411 | NZ_CP010368 |
| *A. parvus* DSM 16617 | APOM01000000 |
| *A. populi* PBJ7$^{T}$ | NZ_NEXX00000000 |
| *A. pragensis* ANC 4149$^{T}$ | LUAW01000000 |
| *A. proteolyticus* 2P01AA | PISJ01000000 |
| *A. puyangensis* ANC 4466$^{T}$ | OANT01000000 |
| *A. qingfengensis* ANC 4671$^{T}$ | NZ_MKKK00000000 |
| *A. radioresistens* DSM 6976$^{T}$ | APQF01000000 |
| *A. rudis* CIP 110305$^{T}$ | ATGI01000000 |
| *A. schindleri* ACE | NZ_CP015615 |
| *A. seifertii* NIPH 973$^{T}$ | APOO01000000 |
| *A. soli* GFJ2 | NZ_CP016896 |
| *A. tandoii* DSM 14970$^{T}$ | AQFM01000000 |
| *A. towneri* DSM 14962$^{T}$ | APPY01000000 |
| *A. ursingii* DSM 16037 | APQA01000000 |
| *A. venetianus* VE-C3 | NZ_CM001772 |

**Supplementary Table S3.** List of oligonucleotides used.

| Primer | Sequence (5'-3') | Reference |
|---|---|---|
| M2a_sc14for | GGGGTCGTATCCGCACCATG | this work |
| M2a_sc14rev | GTTTTTTAATAGGGGATTCTTTAGGCTCAC | this work |
| M2a_sc84for | CCCGAATATCCAAAGATGCCG | this work |
| M2a_sc84rev | GCCTTGTGATGGTTTTGCTTTTGC | this work |
| M2a_sc94for | GGGACCACCGTAATCATAATCATC | this work |
| M2a_sc94rev | GTTCAATGCCAGCTTGTCGC | this work |
| M2a_sc98for | GGAGTAGCAATAATTATAGAGGCAATAC | this work |
| M2a_sc98rev | CCACCCATTGCCTCAACATAC | this work |
| M2a_sc115for | GGCTATCAACTAGAAACTACAGCG | this work |
| M2a_sc115rev | CGTGGCTTACACTCAAATTTCG | this work |
| M2a_sc116for | GACCAGGTTCAATGTGTTCTACG | this work |
| M2a_sc116rev | CTGATCGCATAGATCAGCAAAATCC | this work |
| M2a_sc117for | GCCCCATTTTTAGGTGCATTC | this work |
| M2a_sc117rev | GCATTAGCCCAATGCTACACGC | this work |
| M2a_sc119for | CTCACAAGCCCAAAAAGATACTAAC | this work |
| M2a_sc119rev | GAATAAGTATCTTCTTCCCAGACACC | this work |
| M2a_sc119rev2 | GTACTATGGGTTAGAGCAAGATGG | this work |
| M2a_sc145for | CGCATGATTGAGGCATGGAAC | this work |
| M2a_sc145rev | GCTCTATCACTCGGATGCGC | this work |
| M2a_sc147for | CGCGATTGCAGCTAAGATACC | this work |
| M2a_sc147rev | CCTTCAACCGAGCTTTAGCG | this work |
| M2a_sc187for | TGGAAGCTTCTACTTAGGGCTAAC | this work |
| M2a_sc187rev | CAACTGAAGCTTCAAATGTTGACG | this work |
| M2a_sc190for | CATATCGTGTTGCTATTCTGAACCG | this work |
| M2a_sc190rev | TGCTTTCAAAAGACTTATGAATTAAGTC | this work |
| M2a_sc232for | CTAATACCCAATGAGTCCCCAAAG | this work |
| M2a_sc232rev | GAATAAAGGCTCTCACTCTCAGTTAG | this work |
| M2a_sc165rev | CTACCGATATGCAACAATTGGGTAGAG | this work |
| M2a_sc127for | TCAGTGCGCTTGCCGGATTC | this work |
| M2a_sc167rev | CATCTTAGCAGAGGCAGGACTGT | this work |
| M2a_sc103for | GGTTTTAGATTTTGCAGGGCCTTATG | this work |
| M2a_sc103rev | CCGGATTTTTCAGCCATTGAGGC | this work |
| M2a_sc150for | GTGGAACAATGATAGATTGCCATGCAG | this work |
| M2a_sc150rev | ATATTGGGGAAATGTCGAATTTGAGC | this work |
| M2a_sc176for | ACCTTGGGGAAGATGGGCTATAAAC | this work |
| M2a_sc116for2 | TGTTGCGTTTGTTGCCGCTG | this work |
| M2a_sc188rev | TGCTTTACGTTTTTTACCTTCATCCTTTTTTTG | this work |
| 27for | AGAGTTTGATCCTGGCTCAG | [1] |
| 1492rev | GGTTACCTTGTTACGACTT | [1] |

Reference


1. Lane, D. J. 16S/23S rRNA sequencing in *Nucleic Acid Techniques in Bacterial Systematics* (Eds. Stackebrandt, E., & Goodfellow, M.), p. 115-175. John Wiley & Sons, Chichester, New York. Wiley, ©1991 (1991).

**Supplementary data 1**. The scaffold list of WGS of strain M2a (DDBJ/ENA/GenBank accession VCND00000000). The scaffolds assembled into the plasmid sequences (Fig. 2 and Table 2) are highlighted.

| Chromosomal scaffolds | | | Plasmid scaffolds | IS scaffolds |
|---|---|---|---|---|
| sc_0 | sc_61 | sc_135 | sc_10 | **sc_165** |
| sc_1 | sc_62 | sc_136 | **sc_14** | sc_185 |
| sc_2 | sc_63 | sc_138 | sc_55 | **sc_191** |
| sc_3 | sc_64 | sc_139 | sc_59 | sc_193 |
| sc_4 | sc_67 | sc_140 | sc_65 | sc_194 |
| sc_5 | sc_68 | sc_141 | sc_66 | sc_195 |
| sc_6 | sc_69 | sc_142 | **sc_84** | sc_196 |
| sc_7 | sc_70 | sc_143 | **sc_93** | sc_198 |
| sc_8 | sc_71 | sc_146 | **sc_94** | sc_201 |
| sc_9 | sc_72 | sc_151 | **sc_98** | sc_203 |
| sc_11 | sc_73 | sc_152 | **sc_103** | sc_205 |
| sc_12 | sc_74 | sc_153 | sc_104 | sc_211 |
| sc_13 | sc_75 | sc_155 | sc_109 | sc_212 |
| sc_15 | sc_76 | sc_156 | sc_111 | **sc_213** |
| sc_16 | sc_77 | sc_157 | **sc_115** | sc_215 |
| sc_17 | sc_78 | sc_158 | **sc_116** | sc_217 |
| sc_18 | sc_79 | sc_159 | **sc_117** | sc_218 |
| sc_19 | sc_80 | sc_160 | **sc_119** | **sc_219** |
| sc_20 | sc_81 | sc_162 | **sc_127** | sc_220 |
| sc_21 | sc_82 | sc_163 | **sc_130** | sc_222 |
| sc_22 | sc_83 | sc_169 | sc_132 | sc_225 |
| sc_23 | sc_85 | sc_170 | sc_137 | sc_227 |
| sc_24 | sc_86 | sc_171 | **sc_144** | sc_228 |
| sc_25 | sc_87 | sc_172 | **sc_145** | sc_230 |
| sc_26 | sc_88 | sc_173 | **sc_147** | sc_233 |
| sc_27 | sc_89 | sc_174 | sc_148 | sc_234 |
| sc_28 | sc_90 | sc_180 | sc_149 | sc_235 |
| sc_29 | sc_91 | sc_181 | **sc_150** | sc_237 |
| sc_30 | sc_92 | sc_183 | sc_154 | sc_240 |
| sc_31 | sc_95 | sc_186 | sc_161 | sc_241 |
| sc_32 | sc_96 | sc_199 | sc_164 | sc_244 |
| sc_33 | sc_97 | sc_200 | sc_166 | sc_245 |
| sc_34 | sc_99 | sc_202 | **sc_167** | sc_249 |
| sc_35 | sc_100 | sc_204 | **sc_168** | sc_250 |
| sc_36 | sc_101 | sc_206 | **sc_175** | sc_253 |
| sc_37 | sc_102 | sc_207 | **sc_176** | sc_254 |
| sc_38 | sc_105 | sc_208 | **sc_177** | sc_255 |
| sc_39 | sc_106 | sc_209 | sc_178 | sc_256 |
| sc_40 | sc_107 | sc_210 | sc_179 | sc_259 |
| sc_41 | sc_108 | sc_214 | sc_182 | sc_262 |
| sc_42 | sc_110 | sc_216 | sc_184 | sc_265 |
| sc_43 | sc_112 | sc_221 | **sc_187** | sc_266 |
| sc_44 | sc_113 | sc_224 | **sc_188** | sc_267 |
| sc_45 | sc_114 | sc_226 | sc_189 | sc_270 |
| sc_46 | sc_118 | sc_229 | **sc_190** | sc_271 |
| sc_47 | sc_120 | sc_231 | sc_192 | sc_274 |
| sc_48 | sc_121 | sc_243 | sc_197 | sc_275 |
| sc_49 | sc_122 | sc_248 | **sc_223** | sc_277 |
| sc_50 | sc_123 | sc_251 | sc_232 | sc_282 |
| sc_51 | sc_124 | sc_258 | sc_238 | sc_283 |
| sc_52 | sc_125 | sc_261 | **sc_239** | sc_284 |
| sc_53 | sc_126 | sc_263 | sc_247 | sc_286 |
| sc_54 | sc_128 | sc_269 | sc_264 | |
| sc_56 | sc_129 | sc_281 | **sc_268** | |
| sc_57 | sc_131 | | **sc_278** | |
| sc_58 | sc_133 | | **sc_279** | |
| sc_60 | sc_134 | | **sc_280** | |

**Supplementary data 2.** DNA sequences of genes applied for the phylogenetic reconstructions.

## M2a 16S rDNA

acctagcggcggacgggtgagtaatgcttaggaatctgcctattagtgggggacaacatctcgaaagg
gatgctaataccgcatacgtcctacgggagaaagcaggggaycttcgggccttgcgctaatagatgag
cctaagtcggattagctagttggtggggtaaaggcctaccaaggcgacgatctgtagcgggtctgaga
ggatgatccgccacactgggactgagacacggcccagactcctacgggaggcagcagtggggaatatt
ggacaatgggggaaccctgatccagccatgccgcgtgtgtgaagaaggccttttggttgtaaagcac
tttaagcgaggaggaggctaccgagattaatactcttggatagtggacgttactcgcagaataagcac
cggctaactctgtgccagcagccgcggtaatacagagggtgcaagcgttaatcggatttactgggcgt
aaagcgcgcgtaggtggccaattaagtcaaatgtgaaatccccgagcttaacttgggaattgcattcg
atactggttggctagagtatgggagaggatggtagaattccaggtgtagcggtgaaatgcgtagagat
ctggaggaataccgatggcgaaggcagccatctggcctaatactgacactgaggtgcgaaagcatggg
gagcaaacaggattagataccctggtagtccatgccgtaaacgatgtctactagccgttggggccttt
gaggctttagtggcgcagctaacgcgataagtagaccgcctggggagtacggtcgcaagactaaaact
caaatgaattgacgggggcccgcacaagcggtggagcatgtggtttaattcgatgcaacgcgaagaac
cttacctggtcttgacatagtaagaactttccagagatggattggtgccttcgggaacttacatacag
gtgctgcatggctgtcgtcagctcgtgtcgtgagatgttgggttaagtcccgcaacgagcgcaaccct
tttccttatttgccagcgggttaagccgggaactttaaggatactgccagtgacaaactggaggaagg
cggggacgacgtcaagtcatcatggcccttacgaccagggctacacacgtgctacaatggtcggtaca
aagggttgctacc

## M2a *rpoB*

atggcatactcatataccgaaaagaaacggatccgtaagaattttggtaaattgcctagcgtcatgga
tgctccgtacttgctcgcgattcaagtcgactcgtacagaacattcttacaagatggcaaatcaccaa
aaaaccgcgaagatatcggtctccaagccgcatttcgttcagtttttcctattgaaagttattctggc
aatgctgctttagaatttgttgagtatagtcttggtaagcctgagtttgatgtacgcgaatgtattct
tcgtggctcaacttatgcagcaccaatgcgtgtaaaaattcgtttgatcctgaaagatcgtgaaacga
agtcaattaaagacgtacgtgaacaagaagtctatatgggcgaaatgccattaatgacggataacggt
acctttgtgattaacggtaccgagcgtgtgatcgtgtcacaattacaccgttcaccaggcgtattctt
tgaccacgataaaggcaagactcactcaagtggtaaagtcctttattcagcgcgtatcattccttacc
gtggttcatggttagactttgaattcgatgccaaagacctcgtctatgtacgtattgaccgtcgtcgt
aaattgcttgcgactgtggtgcttcgtgccttgggttatagcaacgaaaacattctcgacatgttcta
cgagaaagtacctgtgtatcttgacatgggtagctaccagattgacctggtgcctgaacgtctgcgtg
gcgaaatggcacaatttgacatcctggacaaggatggcaaggcaattgttgagcaaggtaaacgtatc
aatgcgcgtcatgtacgtcaaatggaagcttcaggtcttgaaaaacttgcagtgcctgatgagtacct
gtatgagcgtatcactgctgaagacatcccattaaaagatggtgatgtgattgcagccaatactgtat
taagccatgaaatcatggtgaaaattgcagaaggcggcgtgaagcagttcaatattctgttcaccaat
gatatcgaccgcggttcattcgttgcagattctctacgtgcagatacgacgagcaatcgtgaagaagc
attggtagaaatctacaaagtgatgcgtccgggcgaaccaccaacaaaagaagctgctgaaaacttat
tcaacaacttgttcttctcttctgaacgctatgacttgtctccagtcggtcgtatgaagttcaaccgt
cgtttgggtcgtccttacgaagtcggtacagaccagaagtcacgtgaagttgaaggcattctctcgaa
cgaagatatcactgatgtattaaaaacattagttgaaatccgtaacggtaaaggtgaagtcgacgata
tcgatcacttgggtaaccgtcgtgttcgttctgttggtgaaatgacagaaaaccaattccgtgtaggt
cttgtacgtgttgaacgtgctgttaaagaacgtttaagccaagctgaaactgacaacctgtctccgca
agatttgatcaatgcgaagccagtagctgctgcaatcaaagaattctttggttcaagccagttgtctc
agtttatggaccaaaacaacccgttatctgagattacgcacaaacgtcgtgtttctgcgcttggtccc
ggcggtttgacgcgtgaacgtgcaggctttgaagtacgtgacgtacatcaaactcactacggtcgtgt
atgtccaattgaaacaccggaaggtccaaacattggtttgatcaactcgctttctgtttatgcgaaat
gtaataacttcggtttcctggaaaccccataccgtaaggttgttgatggtcgtgtaacagatgaagtt
gaatacctgtctgcgattgaagaagtaggcactgtcattgcacaggccgattctgcaatggataaaga
cggtaacttaacagaagagtttgtatctgttcgtcatcaaggtgacttcgtacgtattcctcctgaaa
aagtaacgcatatggatgtatctgctcagcaggtcgtatctgtagcagcgtcactgattccattccta
gaacacgatgatgccaaccgtgcattaatgggttcgaacatgcaacgtcaggcagttccgacgttgat
cgctgacaagccgcttgttggtaccggtatggaagcgaacgtagcacatgactcaggtgtatgtgtga

tcgctcagcgtggtggtcgtatcgagtttgttgatgcgtctcgtgtggttattcgtgtgaatgaagac
gaaatgatcgcaggtgaagcaggtgtagatatctacaacctgatcaaatacacccgttcgaaccagaa
cacttgtatcaaccagaaagttcttgtgaacctgggcgataaagtaggtcgtggtgatgtactggctg
atggtccatcaacagatggcggtgagctggcattgggtcaaaacatgcgcgttgcgtttatgacctgg
aatggttacaactacgaagactcgatcttactttctgaacgtgtacttcaagaagaccgtttaacgtc
tattcatatccaggaattgtcatgtgttgcacgtgataccaaactgggtgcggaagaaatcactgctg
atattccgaacgtaggtgaagctgctctgtctaaactggacgagtcaggtatcgtttacatcggtgct
gaagtaactgctggtgatatccttgttggtaaagtaacccctaaaggtgaaacacagcttactccgga
agaaaaattgctacgtgcaatcttcggtgaaaaagcggctgacgtaaaagactcatctttacgtgttc
catcaggtactaaaggtactgtgattgacgttcaagtgtttacacgtgacggtcttgaaaaagacgaa
cgtgctcaagcaattgaaaaagcgcaattggatgcataccgtaaagacttgaaagaagaattcaaaat
cttcgaagaagctgcacgtgaacgtgtaatccgcctactgaatggccaagagtcgaatggtggcggta
caactaaacgtggcgacaaactgtctgaagacgtgttgtctggtttagagcttgttgatcttcttgaa
attcaaccagttgacgaagcaattgctgaacgtttaactcaaattcaagtgttcttgaaagagaagag
cttcgaaattgatgagaaatttgctgagaaaaaacgcaaactttctacaggcgatgaactgacgactg
gcgtattgaaagtagttaaagtttatcttgctgtgaaacgtcgcatccagcctggtgataagatggcg
ggtcgtcacggtaacaaaggtgttgtatcaaacatcttgccagtagaagacatgccacatgatgccaa
cggtgtacctgttgatatcgtattgaacccgctgggcgtaccatcgcgtatgaacgtgggtcagattc
ttgaaactcacttgggtatggcggcgaaaggtcttggcgatcaaatcgacaagatgatgaaagagcaa
cgtactgtacttgagcttcgtgatttcctagacaagatttacaacaaagttggtggcgagcaagaaga
tcttgatagcttgactgatgaagaaatcttgaaactttctggcaacttgcgtgctggtgtacctttgg
ctactcctgtattcgatggtgctgaagaaggtcagatcaaagagttgttacaacttgcaggcctgtct
agtactggtcagaccgtattatatgatggtcgtactggtgagcgtttcgaccgtccggtaactgttgg
ttacatgtacatgctgaaactgaaccacttggtggatgacaagatgcatgcgcgttcaactggttctt
actctctagttacgcaacagccgcttggtggtaaagcacaattcggtggtcagcgtttcggtgagatg
gaagtctgggcactagaagcttacggtgcagcatatacgctacaagaaatgctgactgtgaaatcgga
tgacgttgaaggccgtacccgtatctacaagaacattgtagatggcaaccattatatggacccgggca
tgcctgaatcgttcaacgtattgaccaaagagatccgttctttaggtatcaacattgaactgaaaaat
ggtgactaa

M2a *gyrB*

atgagttcagaagatcaagctgcttctcaaacagaacaaaccattgaaaaggcttatgattcctctag
catcaaggtattacgtggtttagacgcagtccgtaagcgtccgggcatgtacattggtgacacagacg
atggtacgggcctgcaccacatggtgtttgaagtagtcgacaactcgattgatgaagccttggcaggc
cactgtgatgaaattatagtcaccatccatgaagatgaatcggtttcagtttctgacaatggtcgtgg
tattccgactgacattcaccctgaagaaggtgtgtctgcagcagaggtgattttaaccattctgcatg
ccggtggtaagttcgacgacaacagttataaagtatctggcggtctgcatggcgttggtgtttcagtt
gtaaacgcgctgtctgaaaaactggaattgaccattcatcgtgccggtaaaattcacgaacaagaata
ccgtcatggcgattcacagtatccattaaaagtggtgggcgataccgatagaaccggtactcgtgtcc
gtttctggccaagtgccgagacttttagtcagaccattttaatgttgatattttggcgcgtcgtttg
cgtgaactgtcgttcctaaacgcaggtgtacgtattgtgctgcgtgacgaacgcattaatgccgagca
tgtctttgattatgaaggcggtctgtctgagttcgttaaatatattaacgaaggcaaaactcacctga
atgatattttccatttactgctgcgcaggctgataacggtattacggtagaagtcgcattgcagtgg
aatgattcttatcaggaaaatgtgcgttgctttaccaataacatcccgcaaaaggatgggggtacgca
tttggccggtttccgcgctgcgttgacccgtggtttaaataactacatggacagcgaaaatatcctga
aaaaggaaaaagttgcggtatcgggtgatgatgcacgtgaaggtctgactgccatcgtgtcagtcaaa
gtacctgatccaaaattctcttcacagaccaaagaaaaactggtgtcgagtgaagtgaaaaccgctgt
tgagcaagccatgaacaaggcattttctgaatatttattggaaaatccacaagcagccaaggcgattg
ccggcaagattattgatgcagcacgtgcgcgtgatgcagcgcgtaaagcccgtgaaatgacacgtcgt
aagagtgcgctagatattgccggtcttccaggtaaactggccgattgtcaggaaaaagatccagcttt
gtctgaactgtacctggtcgagggtgattctgcaggtggttcagccaagcaaggtcgtaaccgtaaaa
tgcaggcgattttaccgctgaaaggtaagatcctgaacgtggaacgtgcccgttttgaccgcatgatt
tcctctgctgaagtcggtacgctgattactgcactcggctgtggtattggccgtgaggaatataatcc
ggacaaactgcgttatcacaaaatcattatcatgaccgatgctgacgtcgatggttcgcatatccgta
ccttgctgttaacgttcttcttccgtcaaatgccggaacttgtggaacgtggtcatatttatatcgca

cagccaccattatataagctgaaaaaaggcaagcaggaacagtacatcaaggacaatgatgcactgga
aacttacctgatttcgaatgcgatcgatgagctggagctgcatatcagtgcagaagcacctgcgattc
gtggtgaagcactggcgaatgtgattgccgattatcagacttcacagaagagcttggcgcgtttgacc
cagcgttatcctgcgagtctgcttgatgcgctgctgtcactggaaggctttaagctggatcagctgga
agacaaaacctatgtggaaacatggggtgagaatctgcgtgcagcgattgaagccattcagccaacat
tacgtccagaattaaccctggaacgttttgaaaaagaactggcagatggtcaaaaaactgcaagctgg
ttgccacgtatcacgatctatgtacataacttgccacatagctacctgttagactcagtactgttagg
ttcaagtgaatatgcacgtttattaaagaactcgaagagctggttcagcctgcttgaagagggtgcat
atttacaaaaaggcgatcgcaagattcaggtcagcaatttccatcaggtttggcaacatatcttgcag
gactcacgtcgtggcatgatgatccagcgctataaaggtctgggcgagatgaatgcggaacagttatg
ggaaaccaccatggatccagacaaccgtaacatgctgcaagtgacagttaccgatgcgattgaagcgg
atcgtatgttctcttgcttgatgggcgatgacgtagaaccacgtcgtgctttcattgaagaaaatgcc
ttgaatgcagatattgatgcatag

M2a *recA*

atggatgagaataaaaacaaggcgctcaacgctgccttaagccagattgaaaaacagtttggtaaaaa
tacagttatgcgtcttggtgacaataccgttcaggcagttgaagctgtgtctacaggctctttaacgc
tggatattgcactcggtattggtggtttacctaaaggtcgtatcgtcgagatctatggtcctgaatct
tcaggtaaaaccacaatgaccctgcaagcaattgcagagtgtcagaaagcaggcggtacgtgtgcatt
catcgatgcggaacatgcacttgatcctcaatatgcgcgcaaactgggcgtagatattgacaacctgc
tggtatcgcaaccggatcacggtgaacaggcacttgaaatcgcagacatgctggtacgctctggcgcg
atcgacatgatcgttgtcgactctgttgctgcattgactccgcgcgctgaaattgaaggcgaaatggg
tgactcgcatatgggtctgcaagcccgtttaatgagccaggcactgcgtaaaattactggtaatgcca
agcgttccaactgtatggtgatcttcattaaccagattcgtatgaagattggtgtcatgtttggtagc
cctgaaaccacaactggtggtaacgcactgaaattctatgcctctgttcgcttggacatccgtcgtat
cggccaagtgaaagaaggtgacgagattgttggttctgaaaccaaagtcaaagtcgtgaaaaacaaaa
tggcgcctccgtttaaagaagctttattccagattctttatggcaaaggtgttaaccatctaggtgaa
ttgatcgaccttgctgtacagcaagaaatcgtgcagaaagctggtgcgtggtattcttatcaaggcga
caaaattggccagggtaagaacaacaccatccgttatctggaagagcataaagaaatggctcagacga
ttgaaaaactgattcgtgaccagcttctgactaaagcagtggttgttgaagaagacgatagcaaggaa
gaacctgacttttttagatgcatga

**Supplementary data 3.** Integrases found in strain M2a.

sc_8: 26451-25141, 1311 bp Phage integrase

MAYIKKIYVNYKDCSTQTTLVLPCILTEKGVIISHLRYLAWFNSKSESWKERSCYALKLLLKYINAVPQIENATKLLKAFT
EALIIGTIDPTTRTDALDLYWRPRTVRDTNNLLFHITHYTDFLMLQDEHSTIRVNPFRKATSYEERLNWCAYYHKQANVFL
NHLTKKDLSQQQVRLVGSFNEELIDFEYAVRFPEDQIERLIYLGFEKNGQYDYKSQAITMLMNYGGLRKSEIFHIYTSDIT
LNPNRPQEALVRVYHPELGIAPDPHFKNRREFLLSKTSFKPRNNYPFTERLYAGWKNPLLTSKQGYFEVVFNPPEKAKEFL
LIWANYLKYQRVEPLRSNPHPFAFTNSLGEPETIKNFQRLHKRAVERIGLICKKDHGTTEHGHRHSYGFRARQAGLNQVEI
QKAMHHKSPTSCLVYIKPTLEEVQEKLREIQ

sc_11: 3103-1826, 1278 bp Integrase – P4-like integrase, XerC superfamily

MAKQSNKLTAKTVKNLAYDPESSNKHADGGGLYLFIHKNGSKYWRLDYRRPISQKRNTLALGVYDEVTLEKARLKRDEVKR
MLADGIDPAEERNHHRDELRAKLENTFEKLALEWLKIRELEGKVDRETIRKLNRDILPFIGKLPVTDLSVEQLERDVTNRL
VERGALESARRVKSIMGMVLKLPFKRRLITYNPAYDITLPKPIKGNHNAVVSESELKTMLQKIWRYHAESPRARLRTELAL
KLSAYIYQRPNEIRELLWEHVDFENQRLCFRASKTHQDHIVPLSRQAFDILKQLEDMRTTSAFVFPSVKSPHESMSEDTIR
QALNRLGYKGKHTAHGFRATARTILGEELEYRVDIIEHQLAHTVRDPNGTAYNRTKFLRRRRELMQLWADYLDTLRQGGDV
SVFKPENDENLIKFTKYSVA

sc_18: 39377-40519, 1143 bp Integrase – P4-like integrase, XerC superfamily

MPLTDTECRKAQPKDKQYRLSDSHGLSLIITTKGQKYWNVRVTVHGERKSESLGPYPDLSLKKARELAYELKHRYSRSVLH
EDLKPYFKEVAEDWFNNQKETWSSKHISNVRASLNELYIALANKRINQIQAPEILQIIKKIEARGSLEIAKRTLSRCGMVM
KYAIAHGYRYDNPAGDLVYALKNKRVKNLASLSASEMPEFLRKVRAYPSDAQTHHAIILIMLTGVRVSELLQARWEEFDLE
GRKWDIPEERMKNRLPHRVPLTDMMITELQALRLTHNQELLFPHRLNNKESMRSESILAVIKRSGYAGRMTTHGFRSLFST
VLNESNLFNSDAIERQLAHVPQNRIRSAYNRAQYWDERVKIMEWYGEQVEGWMAQY

sc_32: 4826-3756, 1071 bp Integrase - phage integrase family

MISLGEYPIVSLAEARQKQDEIKSLLANNIDPAVHRQQEKAAMLCDENSFEAIAKEYAADRLKDKSQTYVDAFHRAMEKDI
YKVIGHKNIKDVTSADVLKIMQNTVKRVKSQDNRGTGEVTAIENRKKIGSVMRYAIATLRAENDPTYAVREVIARPDVEHA
RPLSLAERKVFRARIDSYGGAESTVNSILFLFYTMLRTIEVRRLQWSFIDFEERTITFEKQTREQLKKGMRLTKKNRTHVV
PMSEQVYQLLLKQKKLTGRKKYVFEGVYKGGMMPATTINRALQYIMQNVTAHDFRATASTLLNELGYDEKWIETQLAHADE
NKTRASYNHAKYLADRRKMMQDWADIVDGWRG

sc_36: 16302-15112, 1191 bp Putative integrase/recombinase protein - phage integrase family

MLSKLQIDAIKPTSKLQKINDSEMLYLFVSPTGRKTWKVLYTLDGKKNTITLGEYPKVLNAKDARLRRDEIKKQISEGIHP
TEQKRQDKIKDLSDMAFADLIQLYAEKVTPHKRGGRVEIVILNGYIKSFPRLMKKPVNQLGQLDMIQFRDERLKKVKSATV
ARDLGLLSAVFKYARQELRIMTSSPLDDVAKPKQSVSRNRRISQDEIARILEAFKYDGRSQPITKKQQTAWAFLFAIETAM
RASEITGLKWADVYDKHVELELTKNGAARKVPLSKRAIELLSYMRGVDETDVCTLKNKVADGEVGRGGLSAYFTQVVKGKL
KITDLTFHDTRHEAISRMVKNARLPVEVLAKITGHKTISILINTYYNPDIEELADHLHKEDDPDIILFKKFV

sc_43: 4218-6224, 2007 bp Site-specific recombinase, phage integrase family

MSDLHHVLESITIIDGEGNISFKEYSIELLEELNNRIIFKIPERRDGLHQVSFNDNRWVKRDETKVISFASLTQEFEYELK
AIFLSAVFLGRKRNYKKIKWSSMTTKFRILKSFALILMDNNLRSFNDLNKLKSLKLQIIAKSFCDSYDTKRDILNTVCFAL
ETYNFITPETYERLLLSRTDKEAHKNKTLSSYESNSFPIIPDHVLLKTFDAIQNYKKKFFEKYKLWSKYNAKEIQNIKKGN
YKVDNGEYLSYREHDGLNCGNFINFLNQFRKVVIFNTLLFTGMRKDEVKEITNTSLFEDEGIFYITSSLGKTVENRLELSW
ISSQSCNEMLNLLVELNKKVKHRVQAVINTNDPRFSEEYINHLKTNLANEKVFSVNYSLNRCIFDTMGYIKKGEMNKDYSI
FKIALDKHDIAQLEFLDCNYKSTVKSSNNYMVKYEDGDYFNFSPHQFRHTFAYFMITNNLCTIREIKHQFKHLRSAMSFIY
SRRGIYSELINHSKSFDETIKIKSLMGFSNSIAKQQSIGGGVKFILNALNLKDFKYNISVDPIAFNNLDQINTYLIQNKDS
INFLPHGFCMNGSDCSLKSVAEPLSCINCHGYVTTNKNLPFWKGLLEDISAKLSKMYQLPIDRREKFENLILNLEQKQKQL
TEIINALDGKKIQVNEIEVV

sc_43: 19260-17314, 1947 bp Phage Integrase (???) – the N-terminal part is related to an *rpoB* domain.

MPQKNKISNELIKAEYAISYPVHNTIISYDSQGKTKSIFSDMVWDFSSQIAQINRRKAVHFNFINNSNVEVKDKLIYEAKI
ISYGLLYAYTGSNSNKLNQLLDDIVQIRFLISLASTINTTLSNISKNNKLFSLILENISQDKRQKIKLLLSFFKKISAFDS
IYNNHSFTLTVDQFDQLQKLLNSRPLETQKQYLVIPTRLYSILYNKTELILNEYLENKLSIEKFAKKRAKSIKNEDGKIIY
KSRSHKYDHNKEVKDFSVLAKKYKILTKIDLQSYLGKLSEIAIARILMFTGMRMGEILNAPEDCLVELDLYDKKIFIINSY
TSKETSNGAMKATWITCSAVQNAIKVLKSINQINSYESSFKKDVNLENPLFLSRANNLNNSLYHLPVKASVRLNDALNYFD
YDLLVNGEDLNELKLTATQDEIDFYKVQLDVQFPISTHQFRRSLTVYAARSTIVQTPALKAQLKHLTKDMTYHYCNQSELA
PNFIAEPTLVEAYQHEINKYNNDCFLEDVINSTEPLFGASGTRLQNLKNDTNIPVFLLDSKSSLKDIKNGKLFYKRTALGG
CSRQGLRDKIAPLSITACLSCSDAVFSERSVRALTKAKENFYKQMKLFDQDSYFALQLRAEINSIDKVLNKRNIYCEIDNA

sc_48: 17202-18467, 1266 bp Prophage CP4-57 integrase

MPKKSKELSALSVAKIKKTGRHTVGGVDGLCLNVEGNSRVWILRAVVGKRLDKDGKLKPHRRDIGIGSYPEVSLAEARAKA
TELRLQIRSGIDPIAHKQQQLKKLHEQQLRNKTFIECAKIVITNKTRELKNQKHIGQWSSTLETYIYPTLGNLIIGTITKI
DIAEALKPIWIEKNETARRIRGRIETIFDYAKAMGYFVGDNPAAWKGNLEPILGNLKQESRPHPSLPYEQVAEFIQHLRQK
KGISPKALEFTILTACRSGEVFGAKWQEIDFKNKVWIIPKERMKADKEHRVPLSQEAINLLESIQEYTQPQDFIFPAPRNG
GMLSDMSLTTLIKRMHEQKLKENGLGYIDPKQNRVITTHGFRSTFRDWSADKTDYPREVCEHVLAHKLPDEVEAAYLRGAY
LEKRKSLMADWANFCT

sc_66: 17442-16195, 1248 bp Integrase

MKTNDVKSDVNAHSKNTPQNVNIRRKKSVEDIYKALKPKAKEYRQPTDIAGLYCRVQPNGKKSWQYRYKNDQGKWAWIGLG
AYPTVNYVQAREKAEGMLNGTLEIKTQAEIKKIKLQDKDALFSTLMNEWLERKKLTWKAETYRKEKQSIEKHLLSVFGDRE
YATITSREWLNHYTEKQINEGIFNRIEKLISYCYGAYSLAKFKKDLLYNPLEGIRDHLAKGETESMKHVKIHELPEMISLI
RNSSSRPVAIGLELLIHMFPRPGELRQARWEQFDFAEAVWIRPPSMMKRGIEHGIPLSQHVMNLLIELKEISPESEYLFPS
RDNINKPISNLTFNAALNRLGYRGKQNPHGFRHIASTNLNKNFSSKSQVIESALSHLKGGVKGAYDKEAHLEERYEIMEWW
SNYIESLLKH

sc_67: 17866-16685, 1182 bp Phage integrase

MATIRERSGKLIADFRYMGIRCRETTNLEDNAYNRRILKKRLEQLEAEITLGTFEYEKYFPKSKRVDDFKEKRSQQIAVQT
KVPLFKEFTEVWFKQKQVEWRTSYQQKVSIIIKNYLIPAFGNQVLSKIKKSDLLNFRASLAKVTHGKDQTSLKASRINQIM
TPLRMILNDAAERYEFESPYKNINNLKESKIEVTPFSLEEVHKILTTVREDFRPYYTIRFFTGMRTSEIDGLQWKNIDLQR
REIHIREALVNGVLGGTKTYGSDRTIQMNDRVYQAFLQQKSLNNGKSEFVFCNRDGGPLDYRLVNKRVWHPILRFLGLKPR
RAYQTRHTAATLWLSAGENPEWIARQLGHSTTEMLFRVYSRYIPNVTRRDGSAFEAMLERLNTEELAHE

sc_104: 1856-3061, 1206 bp Integrase – P4-like integrase, XerC superfamily

MSLTEIKVRQAKSKEKIYFLADDDGLSLKVEPNGRKSWSYRYSLAGTNKRPRMKLGEYPVMSLKEARSVRDEYKFNNYENK
PLHKKYIKTFQDICEEWLEFKIKNSFEDEPRCGVIQLAKKCLDQDVYPNIGLMPFIEIKRYDLVKIIKKIESRNVKEPVKK
AYSYLNQIYDYAVAMGYCEYNIAHGLHKILVNNKIKKNYPHLNSDELSNFLAKLNQINTDPIIKKALLFKLYTGVRGGELL
LCEPHHFDLDKKIWKIPALHIKQYRRKVILGHDIPDFFVPLSDQALEVVKSALVWSHGEKYVFSSPRNNNKPIHFNTLNLI
IRKMGYTKNELTSHGLRSTFSTILNESGLFQSNWIEAQLSHTDKNKTRASYNHAEYFNQRMEMMQWWGDFIDSCTVN

sc_114: 2257-887, 1371 bp Integrase – P4-like integrase, XerC superfamily

MRIHTVLLNMYPYSIMAKKTIPLSDSKCTGAKPQEKDYSLYDGHGLILFIRKSGSKVWRFKYKRANGKDGLMTLGNFPALS
LKAARDKRRELEELLANGIDPIEHGEIQKAKLNNAYSFEVIARDWHKEYSNTGRWIAHTAERALKNLEDYVFPIIGQQSID
AVKPRDLVKVLRSIEESGYAEVLKKTRQRFSSIFSYAISRGLIEENPAYFLKDVFVKTKKSKHHPQLPLEQLPELLQKLEN
DKGYPITKLCTAFALHTFVRSSEFRFARWSEFDIQKGIWTIPGEREFIPGQKFSSRGAKMKIDHLIPLSPQVIDILKEIHP
YSGMTKNVFPKNGDPQGFISESTINKTLRRIGYDTNKEICGHGFRGMACSALVQSTLFQKEAVEKQMSHQERDQVRLAYTH
QAEYLEERKAMLNWWSDYLEANRVKHISPYDFTQQLLNDDVINFKYAKLAK

sc_148: 4238-2751, 1488 bp Site-specific recombinase, phage integrase family, XerC superfamily

MFKHVGPPLENYRLKINPISVDNKRRSKDVLNHFQGTLSTWLPFSEDELSKLMEYSLFWLEIALPELIKVRDYIDGLDLSN
TSHAPFYSWAPNEEFERLTNITINGINVMSSIRSFQKTSLQSHSKPYVYTWKNQYAIALDHVRNALFILVGLTVGMRAREI
GQIMLDDTYKDKNDEYWINITRFKTTNDPNYQGETEVMPIPYFIGNCIDCFKHLKSLSNFNKEGYLFQSNKSRKVLNNFTS
GQIKLITNEIRQFTGIESPHTHRFRKTVAEILINRSEKNIDLIRMLFGHESYAMTLKYIARNPFMVNAIAEAIEVHFTEDF
HEVVRNIRDESYSGKLAERIANTMISIPEKFVGKQLKLQIMNYVSHMLSSGEIWFIHRSALGSYCIYSGEIEKDDLLPCLL
EKDNLGTSLTPDVNNCQLDCQYCVVLEKSRKAISDNINFYHCVLESTDNIGQKAEAMIRKKLAANEKHLETLDMNHHKVKK
ALKMEVVQL

sc_182: 1322-1915, 594 bp Site-specific recombinase, phage integrase family

MLDLDEYFTTESLLQDQAEAASEEFNHVLQTTGGRISSSVISLSIKLSEYPDQDFPISDYSSYKDDVWLLEFPTGSNPVKI
NFRKEDTKYNGLKRALLYYVIPDFCAFGNIRSYATTRSYNSTFNLFLSYIFHYNHLSPDDPSDIDCISPAILNDALDRAKN
NQDTPSHYCDLFRIIRLWLSLSHQEAIPEEVDQEI

**Supplementary Methods**

**Identification of IS elements**

The known ISs or their close relatives were identified by BLASTn search in the IS Finder database. Then all the scaffold termini where no IS sequence was detected were manually analysed by BLAST search in the WGS of M2a to identify the homologous repetitive sequences in the scaffolds. The scaffold termini showing overlaps with 100% homology to other scaffold termini were manually assembled and the putative coding sequences were examined using BLASTp in the IS Finder database whether they are related to transposases. If this gave positive result, the inverted repeat sequences flanking the coding sequence were identified. In this way, new IS elements that could not be found by BLASTn search against IS Finder database were identified in M2a.

The complete new ISs have been named and submitted into the IS Finder database, while the incomplete ones were indicated as "-related (new)" without new name (Suppl. Table S2a). The copy number of complete ISs was estimated by adding the number of the full length elements found inside the scaffolds to the number of pairs of partial left and right IS ends of the same IS species located at the termini of scaffolds, while the incomplete copies were counted by adding the truncated elements inside the scaffolds to the unpaired partial IS ends at scaffold termini (e.g. if 6 left and 7 right ends of an IS were found at scaffold termini, we counted them as 6 full length and one incomplete copies of this element).

Exact determination of the number of IS types was also ambiguous, because there is no widely accepted consensus on what degree of sequence divergence is needed for two elements to be classified as different types or isoelements of the same type. Furthermore, many ISs are present in M2a genome as slightly different copies, which all differ also from the related prototype ISs available in the database. Typical manifestation of this problem was, when the partial left and right ends of the closely related ISs showed the best homology to different IS elements in the database. For example, 2 left ends and 1 right ends showed the best homology to the IS*6* family element IS*1006* (91-99% similarity), while 1 left and 2 right ends were closer to an other close relative, IS*Our1* (99% similarity). When the partial IS ends located in scaffold termini could not unambiguously be paired due to the above mentioned difficulty, we merged the closely related left and right ends and counted them as parts of slightly divergent isoelements (see the elements of IS*51*-related subgroup of IS*3* family and the three IS*6* family elements, Suppl. Table S2a), thus our data may overestimate/underestimate the number of full/incomplete IS copies, respectively.

The IS content of *A. lwoffii* ZS207 was determined by BLASTn search in IS Finder database and then all the newly identified elements of M2a were also searched in ZS207 chromosome and plasmids by BLASTn. The new elements that are related to those identified in M2a, but their similarity is less than 95% were also named and submitted into the IS Finder database (Suppl. Table S2b).
